# Salmon louse labial gland enzymes: implications for host settlement and immune modulation

**DOI:** 10.3389/fgene.2023.1303898

**Published:** 2024-01-17

**Authors:** Helena Marie Doherty Midtbø, Christiane Eichner, Lars Are Hamre, Michael Dondrup, Linn Flesland, Kristoffer Helland Tysseland, Heidi Kongshaug, Andreas Borchel, Renate Hvidsten Skoge, Frank Nilsen, Aina-Cathrine Øvergård

**Affiliations:** ^1^ Sea Lice Research Centre, Department of Biological Sciences, University of Bergen, Bergen, Norway; ^2^ Sea Lice Research Centre, Department of Informatics, University of Bergen, Bergen, Norway

**Keywords:** arthropod, copepod, development, RNA interference, gene expression, transcriptomics, 5-nucleotidase, trypsin

## Abstract

Salmon louse (*Lepeophtheirus salmonis*) is a skin- and blood-feeding ectoparasite, infesting salmonids. While feeding, labial gland proteins from the salmon louse may be deposited on the Atlantic salmon (*Salmo salar*) skin. Previously characterized labial gland proteins are involved in anti-coagulation and may contribute to inhibiting Atlantic salmon from mounting a sufficient immune response against the ectoparasite. As labial gland proteins seem to be important in the host–parasite interaction, we have, therefore, identified and characterized ten enzymes localized to the labial gland. They are a large group of astacins named *L. salmonis* labial gland astacin 1–8 (LsLGA 1–8), one serine protease named *L. salmonis* labial gland serine protease 1 (LsLGSP1), and one apyrase named *L. salmonis* labial gland apyrase 1 (LsLGAp1). Protein domain predictions showed that LsLGA proteins all have N-terminal ShK domains, which may bind to potassium channels targeting the astacins to its substrate. *LsLGA1* and -4 are, in addition, expressed in another gland type, whose secrete also meets the host–parasite interface. This suggests that LsLGA proteins may have an anti-microbial function and may prevent secondary infections in the wounds. LsLGAp1 is predicted to hydrolyze ATP or AMP and is, thereby, suggested to have an immune dampening function. In a knockdown study targeting *LsLGSP1*, a significant increase in *IL-8* and *MMP13* at the skin infestation site was seen under *LsLGSP1* knockdown salmon louse compared to the control, suggesting that LsLGSP1 may have an anti-inflammatory effect. Moreover, most of the identified labial gland proteins are expressed in mature copepodids prior to host settlement, are not regulated by starvation, and are expressed at similar or higher levels in lice infesting the salmon louse-resistant pink salmon (*Oncorhynchus gorbuscha*). This study, thereby, emphasizes the importance of labial gland proteins for host settlement and their immune dampening function. This work can further contribute to anti-salmon louse treatment such as vaccine development, functional feed, or gene-edited salmon louse-resistant Atlantic salmon.

## 1 Introduction

Salmon louse (*Lepeophtheirus salmonis*) is a blood-feeding ectoparasite infesting the salmonid fish species of the Northern Hemisphere. On susceptible salmonid species, the louse manages to feed on the host mucus, skin, and blood, causing mechanical damages first seen as erosions that develop into small ulcers after the louse starts hematophagous feeding (Brandal et al., 1976; Wootten et al., 1982; Jones et al., 1990; Jonsdottir et al., 1992; Grimnes and Jakobsen, 1996). High parasite loads can, therefore, be detrimental to smaller fish, causing chronic stress that increases the susceptibility to other diseases and disturbing the osmotic balance (Grimnes and Jakobsen, 1996; Finstad et al., 2000; Tort, 2011; Barker et al., 2019). Farming susceptible salmonids such as Atlantic salmon (*Salmo salar*), therefore, requires measures to control this high-fecundity copepod to limit negative impacts on both farmed and wild salmonids. Frequent use of chemotherapeutics to control lice infestations has, however, led to a resistance against most available medical treatments (Aaen et al., 2015). Mechanical and biological delousing methods have, therefore, been developed, unfortunately with an adverse impact on fish welfare and high mortality rates of farmed Atlantic salmon (Overton et al., 2019). Hence, there is a need for novel control measures such as vaccines, functional feeds, or marker-assisted selection in fish breeding. A detailed understanding of the host–parasite interaction between the salmon louse and its salmonid host is vital to achieve this.

To know more on how the salmon louse interacts with its host, investigating lice exocrine glands is important as they secrete factors that may be deposited directly onto the host skin (Øvergård et al., 2016). Especially, the labial glands seem to secrete such factors, as they have secretory ducts extending into the lice mouth tube ending in pores near the mandible teeth. As the mandible teeth are introducing skin debris into the oral cavity when the lice feeds (Kabata, 1974), factors secreted onto these teeth are likely to be deposited directly onto the lice feeding site. The labial gland is, therefore, not developed in the first two lice stages, the nauplius stages that are planktonic and lecithotrophic, whereas it is first seen in the copepodid stage when the lice can attach to a host and start feeding on the host epidermis (Johnson and Albright, 1991; Øvergård et al., 2016). Until now, only five genes with labial gland expression have been described, called *L. salmonis* labial gland proteins (LsLGPs) (Øvergård et al., 2022). Three of these LGPs seem to be highly important during the initial establishing phase, especially LsLGP2 that seemingly dampens inflammatory responses. However, most LsLGPs also seem to be of significance during the two successive chalimus stages, two pre-adult stages, and the adult stage. Now, *LsLGP3* that is likely to dampen cellular responses of the host shows a steady increase in the expression level throughout all the parasitic lice stages, while LsLGP4 that has an anti-coagulant function after the lice starts hematophagous feeding is expressed from the pre-adult I stage (Øvergård et al., 2022).

While the LsLGPs are small, charged proteins not encoding any known protein domains (Øvergård et al., 2022), there is some evidence that the salmon louse exocrine glands also express genes encoding various enzymes. Positive DAB staining and localization of putative heme peroxidase transcripts in salmon louse exocrine glands indicate they have peroxidase activity (Bell et al., 2000; Øvergård et al., 2016; Øvergård et al., 2017). In addition, astacin transcripts have been localized to tegumental (teg) glands (Øvergård et al., 2016), and increased metallopeptidase and trypsin activity have been detected in Atlantic salmon mucus after salmon louse infestation (Firth et al., 2000). Moreover, astacins and trypsins have been identified in secretory/excretory products of dopamine-treated and untreated salmon louse (Fast et al., 2007; Hamilton et al., 2018), suggesting they are either secreted or excreted from the lice gut. In the present study, we, therefore, searched our salmon louse labial gland transcriptomic data for potential secretory enzymes to enable a more thorough analysis of enzymes involved in the host–parasite interaction. Here, genes encoding enzymes belonging to the astacin, serine protease, and apyrase families were identified.

While both astacins and trypsins belong to relatively expanded peptidase families with 44 and 168 genes predicted within the salmon louse genome, respectively, only eight putative apyrases have been identified (Skern-Mauritzen et al., 2021), which are enzymes with 5′-nucleotidase activity hydrolyzing nucleotides such as adenosine tri-, di-, and monophosphate (Smith and Kirley, 2006). Common for all three enzyme families is, however, that they play diverse roles in both parasitic and non-parasitic invertebrates. In addition to being important in developmental processes, both astacins and trypsins are involved in the digestion of proteins for nutrition, tissue degradation for host penetration, and immune modulation, while both trypsins and apyrases are found to prevent clotting during hematophagous feeding (Lun et al., 2003; Gallego et al., 2005; Smith and Kirley, 2006; Park et al., 2010; Baska et al., 2013; Yang et al., 2015). Little is known about these enzymes in the salmon lice, though two astacins are likely to be secreted as part of a mucoid layer covering the lice tegument (Øvergård et al., 2016; Harasimczuk et al., 2017), whereas salmon louse enterocytes express at least five trypsin genes likely to act as digestive enzymes (Kvamme et al., 2004). As nothing is known about these enzymes as part of the labial gland secretome, the present study aimed to characterize the putative labial gland-expressed enzymes and to analyze their potential as immune-modulatory factors conducting gene expression and functional knockdown studies.

## 2 Materials and methods

### 2.1 Rearing and the source of *Lepeophtheirus salmonis*

All experimental procedures were performed in accordance with Norwegian animal welfare legislation (Permit ID: 8589 and 26020). For all experiments, the LsGulen strain of *L. salmonis* was used and maintained on farmed Atlantic salmon according to the work of Hamre et al. (2009). The salmon were hand fed a commercial diet (Nutra Olympic 4.0 mm, Skretting) and reared in seawater with a salinity of 34.5 ppt and a temperature of 8°C–10°C if not otherwise mentioned. Eggs, nauplii, and copepodids were kept in a single-well flow through a system developed by Hamre et al. (2009) with seawater from the same water supply as the fish. Prior to sampling of, or infestation with, planktonic stages, the viability of the larva was checked in a stereo microscope, where an overall low mortality was found.

The following number and groups of animals were collected for ontogenetic analysis in pentaplicate samples, all kept at approximately 9°C. Eggs: 1 egg sac (string) containing approximately 200 eggs. Nauplius I, nauplius II, and copepodids (free-living): approximately 100 larvae. Copepodids 2 days after infestation (dpi) and 4 dpi: 60 larvae. Chalimus I: 30 animals, chalimus II: 20 animals, and pre-adult and adult stages: single animals. Adult females: young were defined as adult females prior to enlargement of the genital segment and the extrusion of the first egg string and mature as females producing egg strings.

Moreover, two additional time series were sampled to analyze the phase prior to and right after host attachment more thoroughly. In the first series, both nauplius I and II were sampled mid stage and planktonic copepodids when they were 4 days old (4 days post molting (dpm)), all kept at 9°C (approximately 100 larvae pooled in each biological replicate). In addition, 4-day-old copepodids incubated at 9°C were allowed to infest Atlantic salmon kept at 12°C and sampled at 6 h, 12 h, and 24 h for 5 days (1–5 dpi). Copepodids were sampled with forceps and placed in RNAlater, with approximately 30 copepodids in each biological replicate.

In the second time series, the planktonic copepodid phase was compared to parasitic copepodids. Egg strings were incubated at 9°C, and both hatching and the molt to copepodids were closely monitored to establish a precise timing of events. Planktonic copepodids were sampled at the age of 0, 1, 2, 3, 4, 5, and 8 days. Copepodids with an age of 3 days were used to infect salmon, and, thereafter, sampled at 1, 2, 3, and 5 dpi (9°C), thus equivalent to a total copepodid age of 4, 5, 6, and 8 days. Copepodids on fish were sampled with forceps, while planktonic copepodids were sampled with a pipette and placed in RNAlater (around 30 cops/biological replicate).

To analyze gene expression in starved lice, 15 adult female lice were collected from Atlantic salmon kept at 10°C. Five lice were analyzed from each time point (biological replicates), sampled directly from the host, and two samples were obtained after 24 and 48 h of starvation in flow-through incubators (9°C), respectively.

Analysis of the labial gland enzyme transcript level was also conducted in lice infesting the lice-resistant pink salmon (*Oncorhynchus gorbuscha*). Pink (201 ± 72 g) and Atlantic salmon (245 ± 21 g) were divided among four separate 1 m tanks, respectively, 24 fish/tank, and maintained at 10°C. After a 14-day acclimatization period, the fish were infested with 200 copepodids/fish to ensure that some lice established on the resistant pink salmon. Copepodids (*n* = 3) were sampled together with the underlying fin tissue at the point of attachment, at 1, 3, and 5 days after infestation from both fish species, where each biological replicate consisted of 2–3 fin/lice samples from the same fish pooled for total RNA isolation.

### 2.2 Total RNA purification and cDNA synthesis

All samples for RNA isolation were collected in RNAlater (Life Technologies), kept at 4°C overnight, and stored at −20°C. All total RNA was isolated with a combined Tri Reagent (Sigma-Aldrich) and RNeasy (QIAGEN) method, as previously described (Øvergård et al., 2010), with DNase treatment performed on column. For ontogenetic analysis, adult female lice were purified with Tri Reagent combined with the RNeasy Mini Kit (QIAGEN), while other life stages of the lice were purified with Tri Reagent in combination with the RNeasy Micro Kit (QIAGEN). The extracted RNA was either kept at −80°C until use, or cDNA synthesis was performed directly. For real-time RT–PCR, cDNA synthesis was carried out using the AffinityScript qPCR cDNA Synthesis Kit (Stratagene) according to the supplier’s recommendations using 200 ng lice total RNA or 1,000 ng salmon total RNA in a 10 µL reaction. Samples were diluted ten or five times for lice and salmon samples, respectively, before storage at −20°C. For PCR, the qScript cDNA SuperMix (Quanta Biosciences) was used, applying 1 µg total RNA.

### 2.3 Identification of labial gland enzymes by RNA sequencing

**FIGURE 1:**
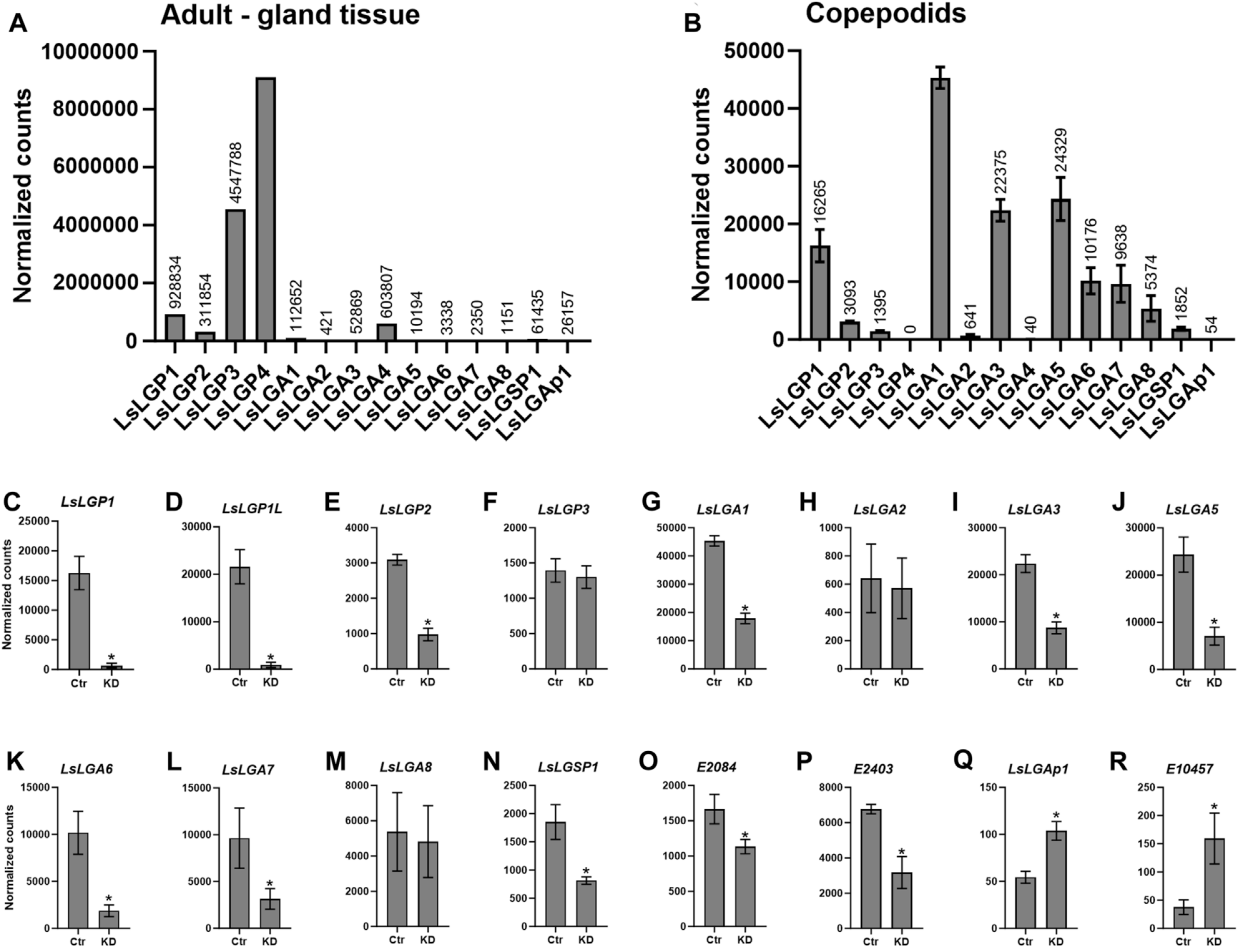
Normalized expression of the labial gland genes in transcriptomic data from **(A)** a sample with a high labial gland content from adult lice and **(B–R)** whole control copepodids 3 dpi from the *LsLGP1/1L* KD study. **(B)** Expression of genes confirmed to be expressed in the labial gland in control animals. **(C–R)** Difference in the transcript level between control (Ctr) and *LsLGP1/1L*-KD animals.

Two approaches were performed to identify possible labial gland enzymes. First, we reanalyzed RNA sequencing data of a sample from adult lice with a high labial gland content previously used to identify the LsLGPs (Øvergård et al., 2022). In short, the region holding the labial gland was dissected out from 20 adult female lice that had been stored in RNAlater (Life Technologies) overnight at 4^○^C. Total RNA was purified, and further library preparation using the TruSeq Stranded mRNA reagents (Illumina) and RNA sequencing (NextSeq 500 instrument (Illumina)) was conducted at the Norwegian Sequencing Centre, Oslo, resulting in 75 bp single-end reads. Data were processed as previously described (Eichner et al., 2018; Øvergård et al., 2022). To identify potential labial gland genes from the obtained data, the labial gland expression was compared to RNA sequencing data obtained for the different developmental stages of the lice, in addition to the gut, gonad, and thoracic feet 1 and 2 expression (Skern-Mauritzen et al., 2021), as previously described (Øvergård et al., 2022) (Supplementary File S1, Figure 1). Sequencing data are available at the NCBI Sequence Read Archive (SRA) (BioProject: PRJNA1008109).

We also utilized the fact that knockdown (KD) of LGP1/1L in copepodids seems to down modulate all other known labial gland-expressed genes (Øvergård et al., 2022), and hence, we compared the transcriptome of LGP1/1L KD copepodids to control copepodids to identify additional genes downmodulated by the KD. For a description of RNAi, see Section 2.7. After the control and KD copepodids had infested fish for 3 days (12°C), around 50 lice were pooled for each biological replicate (N = 3) for RNA isolation. Further library preparation and Illumina sequencing (Illumina HiSeq 4000 (Illumina, Inc., San Diego, CA, United States) were performed at the Genomics Core Facility, University of Bergen, as previously described (Øvergård et al., 2023), with 2 × 75 base pair paired-end reads.

Further analyses were conducted in the web-based platform Galaxy (v 2.11.40.6), maintained by ELIXIR Norway at UseGalaxy.no (Afgan et al., 2018; Tekle et al., 2018). Short reads were mapped to the SeaLice v.0.1 reference genome (LSalAtl2s) with built-in gene models (Skern-Mauritzen et al., 2021), using the RNA STAR alignment tool (Dobin et al., 2013). Aligned reads were counted with featureCounts (v1.0.3), while normalization and differential expression analysis were conducted by DESeq2 (v1.22.1) (Liao et al., 2014; Love et al., 2014). Further DESeq2-normalized counts were normalized to transcript length. Sequencing data are available at the NCBI SRA (BioProject: PRJNA1008109).

### 2.4 *In situ* hybridization

*In situ* hybridization of candidate genes was conducted to confirm labial gland expression. Predicted sequences from the candidate genes were retrieved from Ensembl Metazoa (https://metazoa.ensembl.org/Lepeophtheirus_salmonis/), and gene-specific primers were designed based on these sequences. Single-stranded digoxigenin (DIG)-labeled antisense and sense RNA probes of the selected genes were prepared by *in vitro* transcription using the DIG RNA Labeling Kit (Roche), with purified PCR products that included T7 promoters (TAA​TAC​GAC​TCA​CTA​TAG​GGA​GA) as templates (primers are listed in Supplementary File S1, Table 1). PCR was performed with Q5^®^ High-Fidelity DNA Polymerase as recommended by the supplier (New England Biolabs), applying M13 forward and reverse primers (cycles were initial denaturation at 98°C for 30 s, 30 cycles of 98°C for 10 s, 55°C for 30 s, and 72°C for 30 s/kb, and a final extension for 2 min at 72°C). The products were purified with the GenElute PCR Clean-Up Kit (Sigma-Aldrich). For the highly similar astacin genes, the PCR products were cloned and sequenced as described in Section 2.5, and plasmids were purified from overnight cultures of selected colonies using the GenElute Plasmid DNA Miniprep Kit (Sigma-Aldrich). The plasmids were further used as a template for PCR, and PCR products were purified as described above and added to the DIG-labeling reaction.

**TABLE 1:** Overview of the labial gland enzyme sequences. The length of the obtained nucleotide (nt) sequence after RACE is given, so are the length of the complete ORF-encoded protein (aa) and the mature sequences (in parentheses). The putative mass for the predicted mature proteins is given in kilodalton (kDa).

Name	ENSEMBL stable ID	Accession no.	nt	aa	kDa	Domain
LsLGA1	EMLSAG00000009545	OR504246	1499	348 (329)	37.8	SP—Astacin—2xShK
LsLGA2	EMLSAG00000000817	OR504244	1351	348 (329)	37.6	SP—Astacin—2xShK
LsLGA3	EMLSAG00000002095	OR504245	1272	348 (329)	37.2	SP—Astacin—2xShK
LsLGA4	EMLSAG00000000710	OR504243	1329	348 (329)	37.3	SP—Astacin—2xShK
LsLGA5	EMLSAG00000008402	OR504248	1110	332 (311)	36.5	SP—Astacin—1xShK
LsLGA6	EMLSAG00000006562	OR504249	1441	353 (333)	37.5	SP—Astacin—2xShK
LsLGA7	EMLSAG00000010888	OR504247	1661	473 (454)	51.3	SP—Astacin—4xShK
LsLGA8	EMLSAG00000010512	OR504250	1066	310 (292)	34.1	SP—Astacin—1xShK
LsLGSP1	EMLSAG00000010949	OR504241	1262	388 (368)	41.0	SP—Serine protease
LsLGAp1	EMLSAG00000011718	OR504242	1836	556 (537)	60.2	SP—Apyrase

LsLGA, *Lepeophtheirus salmonis* labial gland astacin; LsLGSP1, *Lepeophtheirus salmonis* labial gland serine protease 1; LsLGAp1, *Lepeophtheirus salmonis* labial gland apyrase 1; SP, signal peptide; ShK, *Stichodactyla helianthus* K channel toxin-like domain.

Copepodid, chalimus I, and adult *L. salmonis* were fixed in phosphate-buffered 4% paraformaldehyde (pH 7.4) for 24 h at 4°C. Subsequently, specimens were processed with the Histokinette 2000 (Reichert-Jung), where they were washed in PBS, dehydrated through a graded ethanol series, and embedded in paraffin wax. Sections, 4.0 μm thick, were cut with a Leica RM 225 microtome (Leica Microsystems). *In situ* hybridization was performed according to the work of Dalvin et al. (2013), with some modifications as described earlier (Tröße et al., 2014). Hybridization with sense probe was performed as negative control. Additionally, the proteinase K treatment was prolonged to 18 min. Antisense and sense probe were applied to adjacent sections at each round of hybridization, to control for unspecific hybridization. Adjacent sections were also stained with hematoxylin (Shandon Instant Hematoxylin, Thermo Scientific) for 2.5 and 1.5 min with 1% erythrosine (Certistain, Merck). HE-stained sections were mounted in Histomount (Invitrogen).

### 2.5 Cloning and sequencing

When genes were confirmed to be expressed in the labial glands, primers for RACE were designed from the predicted sequences retrieved at Ensembl Metazoa (Supplementary File S1, Table 1). RACE (5′ and 3′) was performed for all genes using the SMARTer™ RACE cDNA Amplification Kit according to the supplier’s instructions (Clontech). Total RNA from parasitic copepodids and adult lice were used for RACE-ready cDNA preparation, and RACE PCR products were further cloned using the TOPO TA Cloning^®^ Kit for Sequencing (Invitrogen). Colonies were used as templates for PCR reaction using the Q5^®^ High-Fidelity DNA Polymerase kit according to the supplier’s recommendation (New England Biolabs), as described in Section 2.4. PCR products were further purified by ExoSAP-IT (Affymetrix) and sequenced using the BigDye Terminator v3.1 Cycle Sequencing Kit from Applied Biosystems. Sequencing was completed on an ABI prism 7700 automated sequencing apparatus at the University of Bergen sequencing facility. The open reading frame (ORF) was further confirmed by one directly sequenced PCR product as described above.

Sequences were assembled and translated using Vector NTI Advance 9 software (Invitrogen). The ORF was blasted using NCBI BLAST form (Altschul et al., 1990) and aligned with similar sequences in Clustal Omega to look for sequence conservations (Sievers et al., 2011). The location of domains was predicted by InterPro (Paysan-Lafosse et al., 2023) and signal peptides by the SignalP 5.0 server (Almagro Armenteros et al., 2019). ProtParam was used to compute molecular weights (Gasteiger et al., 2005), while potential sites for GPI-anchoring were analyzed in NetGPI-1.1 (Gislason et al., 2021). Glycosylation sites were predicted in NetNGlyc 1.0 and NetOGlyc 4.0 (Gupta and Brunak, 2002; Steentoft et al., 2013). Protein structure was predicted in AlphaFold2 (Jumper et al., 2021).

### 2.6 Real-time RT–PCR

Real-time RT–PCR was performed with 1x PowerUp™ SYBR Green Master Mix (Thermo Fisher Scientific), 500 nM forward and reverse primers (Supplementary File S1, Table 1), and 2 µL diluted cDNA in 10 µL reactions. Samples were run in duplicate on the QuantStudio 3 Real-Time PCR System under the conditions of 50°C for 2 min, 95°C for 2 min, 40 cycles of 95°C for 15 s, and 60°C for 1 min, followed by a melt curve analysis at 60°C–95°C). A five-point standard curve of 4-fold dilutions was made for each assay to calculate PCR efficiencies, given by the equation E% = (10^1/slope^—1) x 100 (Radonic et al., 2004). The relative differences in the threshold cycle between the target gene and the reference genes (ΔCT) and expression relative to a calibrator (ΔΔCT) were calculated, transformed by the equation 2^−ΔΔCT^ (Pfaffl, 2001). Statistical analysis was performed by GraphPad prism 8.0.1 (GraphPad Software).

Student’s t-tests were used to test for differences between lice on pink and Atlantic salmon, as only two groups were compared (results were considered significant for *p* < 0.05). A one-way ANOVA was used to test for differences when more than two groups were compared in the starvation and KD study with Bonferroni’s multiple comparisons *post hoc* test (results were considered significant for *p* < 0.05), as the data met the requirements of equal variance.

### 2.7 RNA interference and infestation studies

RNA interference (RNAi) with subsequent infestation studies was performed to analyze the immune-modulatory capability of the labial gland proteins *in vitro*. RNAi on salmon louse nauplii was performed as previously described (Eichner et al., 2014). In short, long double-stranded RNA for the selected genes was produced using the MEGAscript RNAi Kit (Ambion) according to the supplier’s instructions using primers listed in Supplementary File S1 (Table 1). As LsLGA1–4 are highly similar, all four genes were knocked down simultaneously. For the other genes, only one gene was targeted per animal. Around 60–100 nauplius I larvae, all from the same egg string, were incubated overnight (17 h) in 150 µL of seawater containing 20 ng/µ1 control or labial gland gene dsRNA. Thereafter, all animals within a treatment group were pooled and kept in flow-through incubators until the copepodid stage was reached.

In a series of experiments, knockdown copepodids were allowed to infest Atlantic salmon (average weight around 150 g, *n* = 6–8) kept in single tanks at 12°C (Hamre and Nilsen, 2011). In addition, each infestation experiment included an untreated group of fish and a group of fish infested with control lice. Around 80 copepodids/fish were added to each fish tank, and at 3 days after infestation (3 dpi), scaled skin samples were taken at and away from the lice attachment site 3 days after infestation (3 dpi). Skin samples from the attachment site were kept as small as possible, with a size of around 5*5 mm and two attachment site samples from each fish to ensure enough tissue for further processing. The knockdown of each target gene was repeated twice with equal results.

## 3 Results

### 3.1 Identification of labial gland enzyme genes

To identify possible labial gland enzymes, the labial gland transcriptome was analyzed by Illumina sequencing of a sample with a high gland content. From this dataset, five putative labial gland enzymes were identified (Figure 1A). One of these genes was predicted to be a serine protease (EMLSAG00000010949), while the other was predicted to be an apyrase (EMLSAG00000011718), hereafter called *L. salmonis labial gland serine protease* (*LsLGSP*) *1* and *L. salmonis labial gland apyrase* (*LsLGAp*) *1*, respectively. The last two genes were predicted to be astacins (EMLSAG00000002095 and EMLSAG00000010512), hereafter called *L. salmonis labial gland astacin* (*LsLGA*) *3* and *8*, respectively. Moreover, three additional astacin genes that are highly similar to *LsLGA3* were identified within the salmon louse genome (EMLSAG00000009545, EMLSAG00000000817, and EMLSAG00000000710). These three genes were also included in further analyses, even though EMLSAG00000009545 has previously been shown to be expressed by a special tegumental type 1 (teg 1) gland and named *L. salmonis* astacin (LsAst) 2 (Øvergård et al., 2016). EMLSAG00000009545 (LsAst2) is, hereby, renamed as LsLGA1, and EMLSAG00000000817 and EMLSAG00000000710 are, hereafter, called LsLGA2 and LsLGA4, respectively (Table 1).

We also analyzed the transcriptome of copepodids to identify labial gland enzymes mainly expressed in younger parasitic stages. The genes were identified by investigating genes regulated by *LsLGP1/1L* knockdown in copepodids, previously shown to downregulate all the labial gland genes identified so far (Øvergård et al., 2022). From these data (Figures 1B–R), the KD of *LsLGP1* and *2*, *LsLGSP1*, *LsLGA1*, and *LsLGA3* identified from the labial gland transcriptome of adult females was confirmed to be downregulated in *LsLGP1/1L*-KD animals. Moreover, three additional astacin genes were found to be downregulated (EMLSAG0000008402, EMLSAG0000006562, and EMLSAG0000010888), named *LsLGA5–7* (Figures 1J–L), respectively, in addition to a protein disulfide isomerase-2 (EMLSAG00000002084) (Figure 1O) and a putative glutamate dehydrogenase (EMLSAG00000002403) (Figure 1P) gene. Interestingly, two genes were found to be upregulated upon *LsLGP1/1L*-KD, namely, the *LsLGAp1* and an astacin gene (EMLSAG000000010457) (Figures 1Q–R).

**FIGURE 2:**
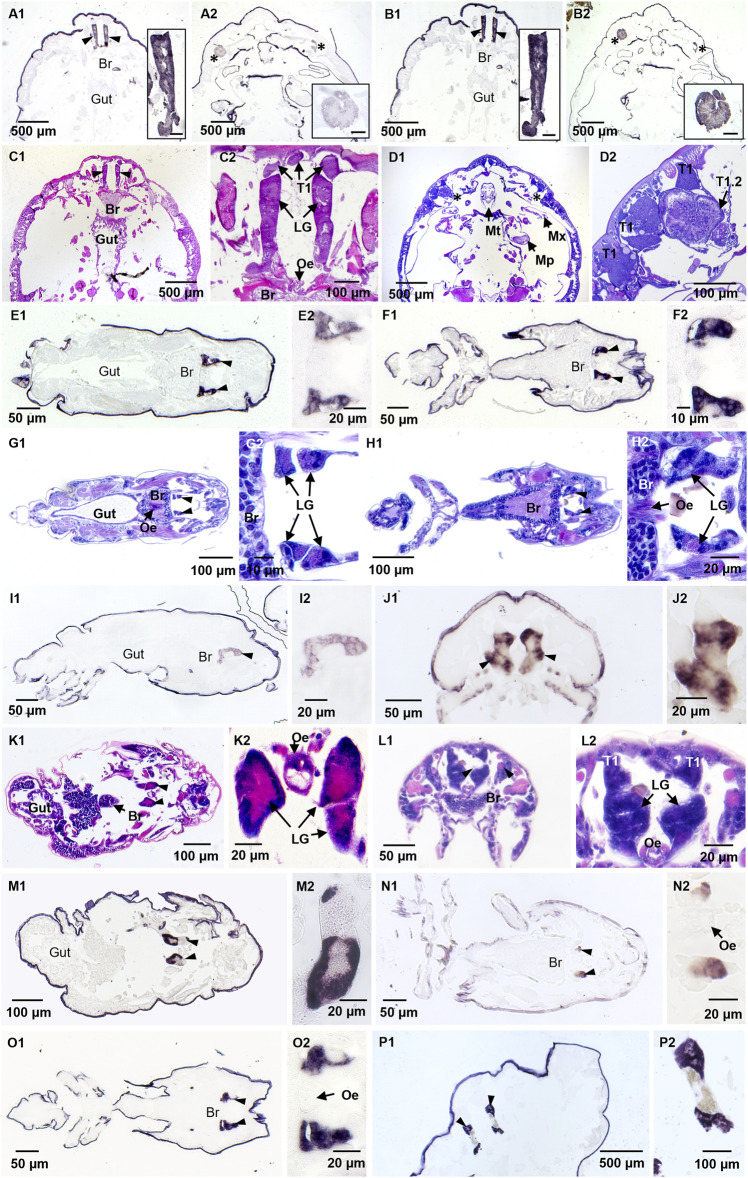
Localization of *Lepeophtheirus salmonis* labial gland enzyme transcripts by *in situ* hybridization and HE staining of adjacent sections. Adult female specimens were treated with antisense probes for LsLGA1 **(A1, A2)**, LsLGA4 **(B1, B2),** and LsLGAp1 **(P1, P2)**; **(C1–D2)** the histology in sections with the labial gland **(C)** and a tegumental type 1 gland subtype **(D)**. Copepodid specimens were treated with antisense probes for LsLGA2 (**E1**, **E2**), LsLGA3 **(F1, F2)**, LsLGA5 **(I1, I2)**, LsLGA6 **(J1, J2)**, LsLGA8 **(N1, N2)**, and LsLGSP1 **(O1, O2)**. Chalimus I specimens were treated with antisense LsLGA7 probe **(M1, M2)**. Histology of **(E)** is shown in **(G)**; while the section stained in **(H)** is adjacent to **(F, O)**, the section in **(K)** is adjacent to **(M)**, the section stained in **(L)** is adjacent to **(J)**. Specific staining of labial gland enzymes is seen in labial glands (LGs, marked with arrowheads) found anterior to the central ganglia (Br) often with the esophagus (Oe) seen medially. A subtype of tegumental type 1 glands (T1.2, marked with asterix) was also stained by LsLGA1 **(B)** and LsLGA4 **(D)** probes. No staining was detected in the gut or the ordinary tegumental type 1 gland (T1). Insets show higher magnification of the stained glands. The scale bar in insets in **(A–D)** is 50 μm. Non-specific staining is seen in the lice cuticula in all pictures.

To confirm the labial gland expression of the identified genes, *in situ* hybridization was performed applying probes complementary to all genes with a potential of being involved in the host–parasite interaction: the astacins, the serine protease, and the apyrase. However, the astacin shown to be upregulated upon *LsLGP1/1L*-KD was not included, as it displays an overall low expression level (Figure 1M). All genes were found to be localized to the labial gland secretory unit, with no staining detected in the reservoir part of the gland from where the large secretory duct protrudes (Figure 2). *LsLGA1* and -*4* transcripts were, however, also detected in the special teg 1 gland (Figures 2B, D), as previously shown for *LsLGA2* (Øvergård et al., 2016). The other *LsLGA* genes were analyzed only in copepodid and chalimus specimens and did not show any staining in teg 1 glands; however, it is not known if this teg 1 gland subtype is developed in these instars (Øvergård et al., 2016). No staining of glands or other tissues was seen in the nearby sections with sense probes added as a negative control (results not shown), but an unspecific staining of the cuticula was seen in both sense and antisense treated sections (Figure 2).

### 3.2 Sequence analysis

After the selected labial gland enzyme transcripts were confirmed to be localized to the labial glands, RACE was performed to verify the sequences predicted from the salmon louse genome. With the complete ORF of the gene, subsequent bioinformatics analysis was conducted to add further functional implications (Table 1, Supplementary File S2). Signal peptides were predicted within the deduced sequence of all enzymes, as were amino acids (aa’s) important for enzymatic activity.

#### 3.2.1 Astacin

The eight astacin metallopeptidase domains (PF01400) were quite similar in size, and all were found to contain the conserved zinc-binding and catalytic site consensus sequence (HExxHxxGxxH) immediately followed by a family-specific glutamate as part of the consensus motif ExxRxDRD (Supplementary File S2). In the C-terminal of the astacin domains, all genes encode a short linker sequence rich in threonine and proline followed by either one, two, or four ShK domains (PF01549) (Table 1). Blast searches with the astacin domains only showed a high resemblance mostly to invertebrate astacins, both insects and crustacean, besides LsLGA8 that was found to have the highest similarity to astacin sequences from the primitive ray-finned fishes *Polyodon spathula* and *Acipenser ruthenus* annotated as high choriolytic enzyme 1-like.

#### 3.2.2 Serine protease

For LsLGSP1, a putative trypsin-like domain (PF00089) was found. All serine proteases have an active site serine that, together with a histidine and an aspartic acid, forms a catalytic triad that catalyzes the hydrolysis of amide bonds (Muhlia-Almazan et al., 2008). The catalytic triad residues, His, Asp, and Ser, were found to be conserved in LsLGSP1 (Supplementary File S2). The specificity pocket of LsLGSP1 was found to consist of Asp and two Gly, further indicating that it may be a trypsin (Perona and Craik, 1995). BlastP searches (NCBI) showed that LsLGSP1 has high resemblance to several invertebrate genes annotated as serine proteases, chymotrypsins, and trypsin-like proteins. Looking specifically into invertebrate species with more functional data available, LsLGSP1 was found to have the highest resemblance to *Ixodes scapularis* serine proteinase stubble and *Caenorhabditis elegans* serine protease svh-1. Limiting the blast search to vertebrate species, LsLGSP1 showed the highest resemblance to type II transmembrane serine proteases, particularly hepsin, prostasin, and matriptase in various fish species.

#### 3.2.3 Apyrase

In the LsLGAp1 sequence, a 5′-nucleotidase/apyrase domain was predicted following the signal peptide. The apyrase domain was predicted to contain an N*-*terminal metal ion-binding and catalysis domain and a C-terminal nucleotide substrate-binding domain (Supplementary File S2). Within the N-terminal domain, aspartic acid, asparagine, and histidine residues known to take part in binding the two zinc ions needed for catalysis (Heuts et al., 2012; Knapp et al., 2012) were found to be conserved in LsLGAp1. In addition, the histidine and aspartate that form a catalytic Asp–His dyad were found, as where cysteines known to be involved in intramolecular disulfide bridge formation. BlastP searches (NCBI) with LsLGAp1 showed the highest resemblance to genes annotated as snake venom 5′-nucleotidase in various *Daphnia* species, followed by various 5′-nucleotidases, including a fish ecto-5′-nucleotidase (CD73), with 56% similarity to human CD73.

### 3.3 Labial gland enzyme transcript levels during salmon louse development

**FIGURE 3:**
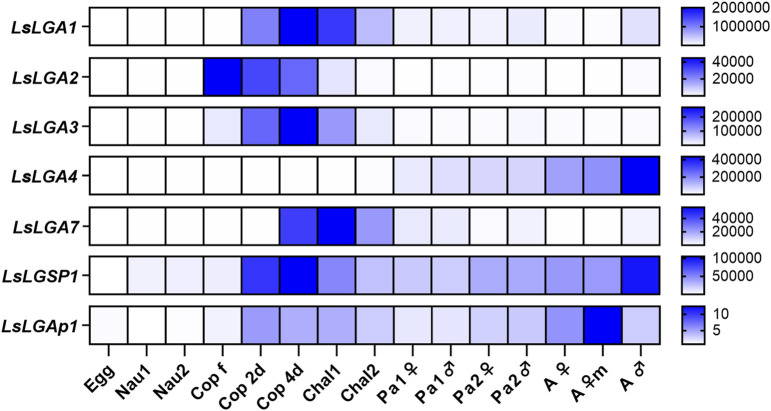
Heat map showing the average transcript levels of the labial gland enzymes (2^−ΔΔCT^) during development from maturing embryos within egg sacs until fully mature adult female and male lice (N = 5). Expression levels are related to *LsEF1α* and *LsADT3* reference genes (∆Ct) and further to the transcript level in eggs (∆∆Ct). Abbreviations: egg, fertilized egg sacs; nau, nauplius; cop free, planktonic copepodids; cop 2d and 4d, copepodids 2 and 4 days after infestation; chal, chalimus; pad, pre-adult; ad, adult lice; m, mature; LsLGA, *Lepeophtheirus salmonis* labial gland astacin; LsLGSP1, *Lepeophtheirus salmonis* labial gland serine protease 1; and LsLGAp1, *Lepeophtheirus salmonis* labial gland apyrase 1.

We further analyzed the expression levels of the labial gland enzymes during the salmon louse lifecycle (Figure 3). *LsLGP1-4* and -*7* were selected for analysis, in addition to *LsLGSP1* and *LsLGAp1*. All genes were found to be expressed mainly in the parasitic life stages and only marginally expressed in eggs and planktonic stages. Moreover, all transcript levels were at their highest either at the newly attached copepodids and/or adult lice stage as previously seen for the LsLGPs (Øvergård et al., 2022). While *LsLGA1-3* and -*7* were primarily expressed in the early larval stages, *LsLGA4* expression gradually increased during the pre-adult and adult stages. Both *LsLGSP1* and *LsLGAp1* displayed a more constitutive expression level during the parasitic stages, most evident for *LsLGAp1*.

**FIGURE 4:**
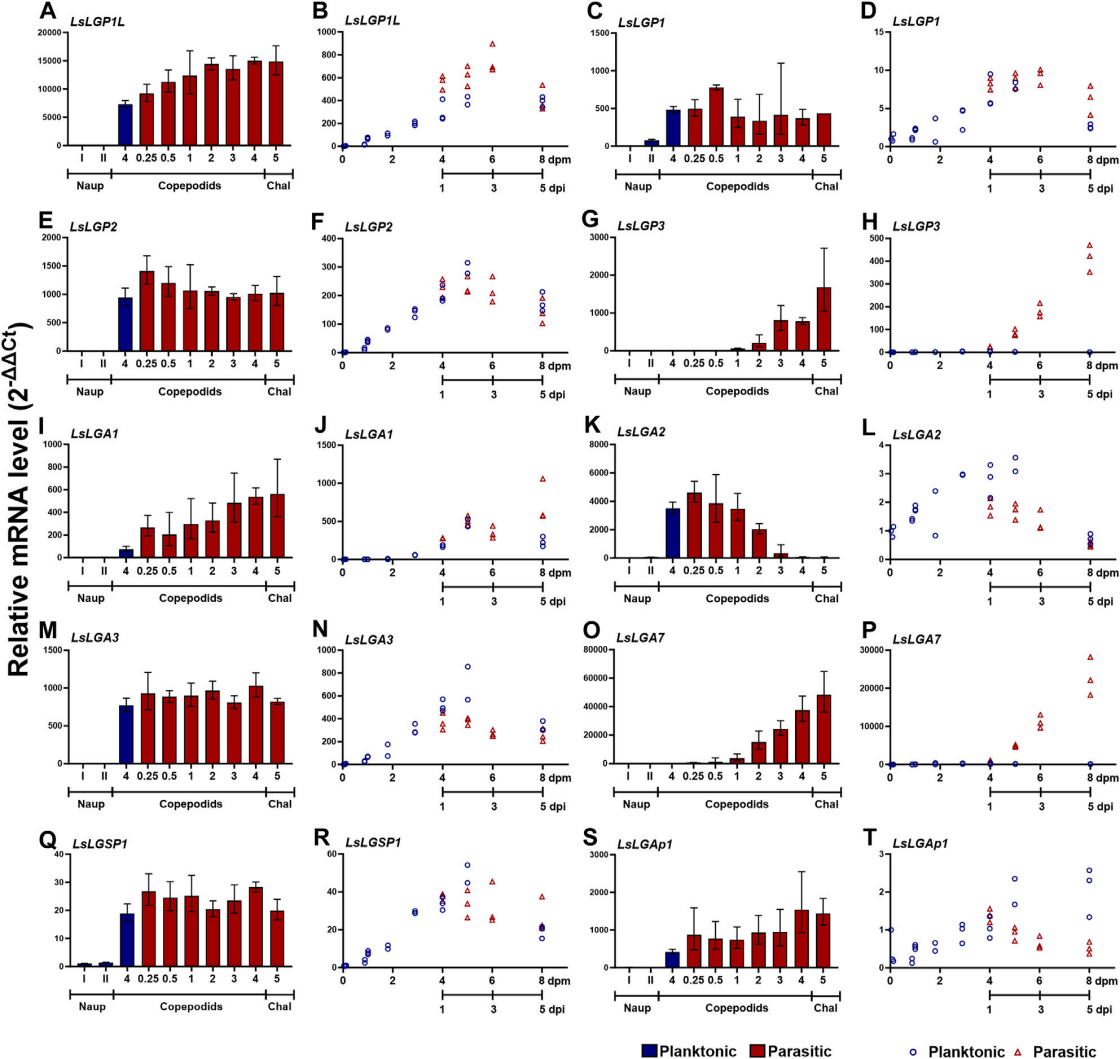
Transcript levels (2^−ΔΔCT^ ± SD) of labial gland genes in two copepodid time series. **(A, C, E, G, I, K, M, O, Q, S)** Expression in nauplius (naup) to chalimus I (chaI) (N = 3) at the medium nauplius I and II stage and planktonic copepodids 4 days after dpm and 0.25, 0.5, 1, 2, 3, 4, and 5 day after infestation (dpi). **(B, D, F, H, J, L, N, P, R, T)** Expression in planktonic copepodids from 0–8 dpm and from parasitic copepodids sampled at 1–5 dpi, with the same relative age as the planktonic copepodids sampled at 4–8 dpm. Expression levels are related to *LsEF1α* and *LsADT3* reference genes (∆Ct) and further to the transcript level in nauplius I or copepodid 0 dpm (∆∆Ct). Abbreviations: LsLGP, *Lepeophtheirus salmonis* labial gland protein; LsLGA, *Lepeophtheirus salmonis* labial gland astacin; LsLGSP1, *Lepeophtheirus salmonis* labial gland serine protease; and LsLGAp1, *Lepeophtheirus salmonis* labial gland apyrase.

To attain a more complete overview of the expression during the copepodid stage, we analyzed additional sampling series that compared the expression in nauplii, planktonic, and parasitic copepodids more closely. In addition to the transcript levels of the selected labial gland enzymes, we also analyzed the transcript level of the previously identified LsLGPs on these samples looking only at genes displaying a relatively high expression level at the copepodid stage. As previously indicated (Øvergård et al., 2022; Figure 3), the activation of labial gland gene expression occurred after molting to copepodids (Figure 4). The expression was, however, in this dataset found to be increased already in planktonic copepodids, and similar transcript levels were detected for most genes between planktonic and parasitic copepodid of the same relative age (Figures 4B, D, F, J, L, N, R, T). Interestingly, induction of two transcripts was dependent on host attachment, namely, *LsLGP3* and *LsLGA7* (Figures 4G, H, O, P). Moreover, *LsLGA2* expression was found to decrease as a response to host attachment (Figure 4L), as indicated in the initial ontogenetic study (Figure 3B).

### 3.4 Labial gland enzyme transcript level during starvation

**FIGURE 5:**
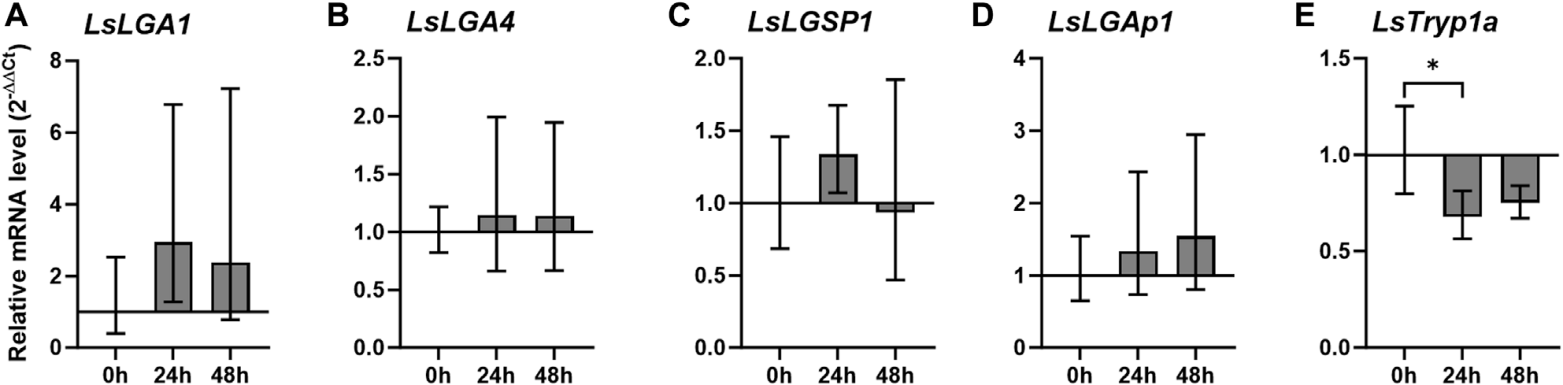
Relative transcript level (2^–ΔΔCT^ ± SD) of **(A–E)** Lepeophtheirus salmonis labial gland genes in adult females at 0, 24, and 48 h (h) after removal from the host (N = 5), where LsTryp1a was analyzed as an example of a gut-expressed gene. Expression levels are related to *LsEF1α* and *LsADT3* reference genes (ΔCt) and further to the transcript level 0 h after removal from the host (ΔΔCt). Abbreviations: LsLGA, *Lepeophtheirus salmonis* labial gland astacin; LsLGSP1, *Lepeophtheirus salmonis* labial gland serine protease 1; LsLGAp1, *Lepeophtheirus salmonis* labial gland apyrase; and LsTryp1, *Lepeophtheirus salmonis* trypsin 1.

We further analyzed the transcript level of the selected labial gland enzymes during starvation, including only genes that were relatively highly expressed at the adult stage. No significant differences were seen for the labial gland enzymes when lice were incubated off the host for 48 h, and however, a gut-expressed trypsin, *LsTryp1a,* was significantly downregulated 24 h after taken off the host (Figure 5). A decrease in *LsTryp1a* was also seen at 48 h, though not found to be significant.

### 3.5 Labial gland enzyme transcript level in lice infesting the resistant pink salmon

The pink salmon is, for unknown reasons, more or less resistant to Pacific salmon louse after it reaches a certain size (Jones, 2011). Several factors can enable resistant salmonids to reject salmon louse, where a lack of immune modulation could be one such factor. We, therefore, analyzed whether the labial gland gene expression was sustained after infestation of pink salmon at levels similar to *L. salmonis* on Atlantic salmon. The transcript level of previously identified LsLGPs were also included in this analysis, so as to give a broader perspective on the salmon louse labial gland expression upon pink salmon infestation.

**FIGURE 6:**
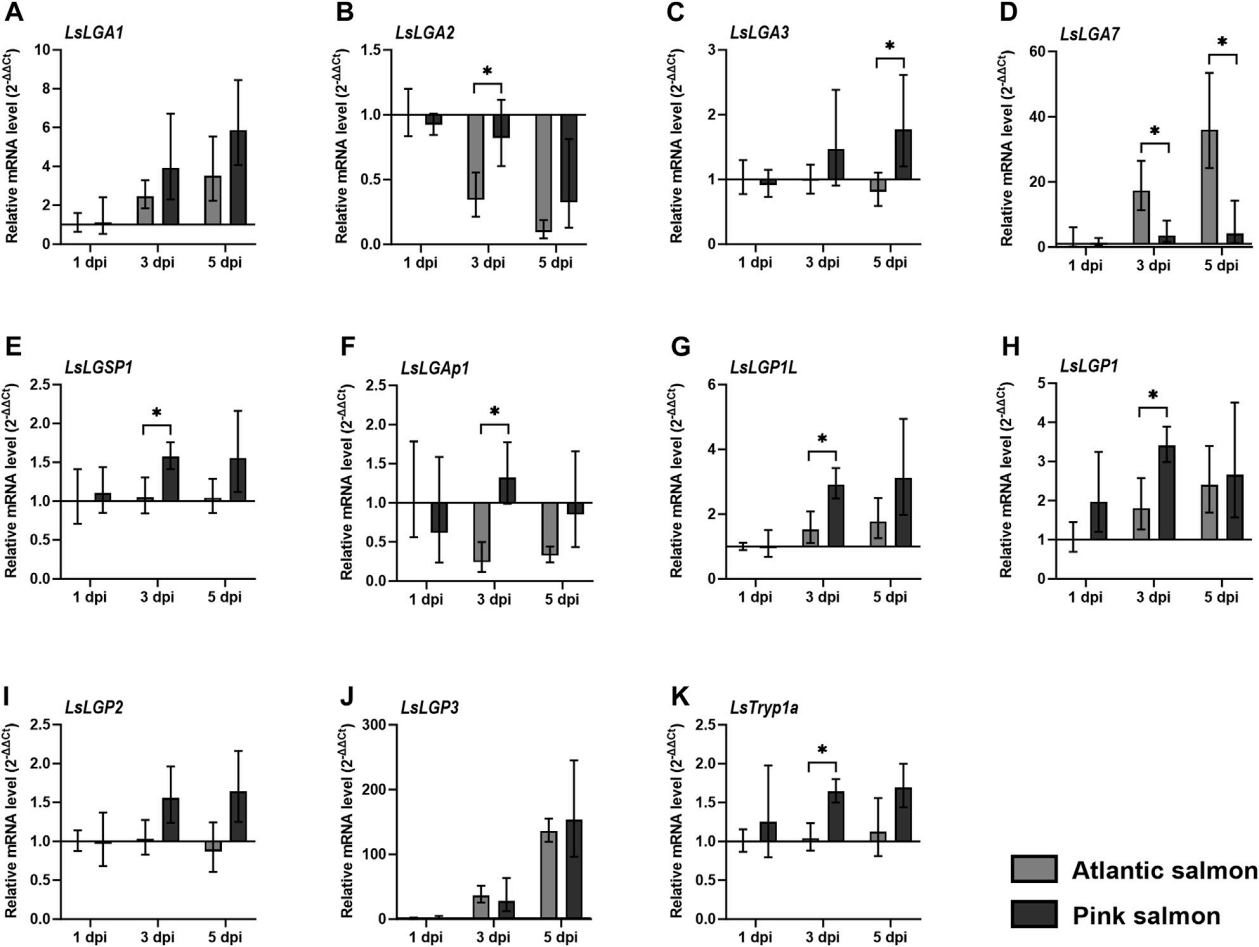
Relative transcript level (2^–ΔΔCT^ ± SD) of **(A–K)** selected *Lepeophtheirus salmonis* genes in lice attached to Atlantic and pink salmon fins (N = 5), for 1, 3, and 5 days after infestation (dpi). Expression levels are related to *LsEF1α* and LsADT3 reference genes (ΔCt), and expression in both groups of lice was related to the transcript level in copepodids on Atlantic salmon at 1 dpi (ΔΔCt). * indicates significantly different expression between lice on Atlantic (*Salmo salar*) and pink salmon (*Oncorhynchus gorbuscha*) (*p* ≤ 0.05). Abbreviations: LsLGP, *Lepeophtheirus salmonis* labial gland protein; LsLGA, *Lepeophtheirus salmonis* labial gland astacin; LsLGSP1, *Lepeophtheirus salmonis* labial gland serine protease 1; LsLGAp1, *Lepeophtheirus salmonis* labial gland apyrase 1; and LsTryp1a, *Lepeophtheirus salmonis* trypsin 1 a.

Overall, most genes displayed similar or increased transcript levels in lice on pink salmon compared to lice on Atlantic salmon (Figure 6), where *LsLGA3*, *LsLGSP1*, *LsLGAp1, LsLGP1L*, and *LsLGP1* were significantly elevated at 3 dpi (Figures 6B, E–H). *LsLGA3* also displayed a higher transcript level upon pink salmon infestation at 3 dpi, but this was only significantly elevated at 5 dpi (Figure 6C). On the other hand, *LsLGA7* expression was not induced in lice infesting pink salmon (Figure 6D). We also analyzed the level of the gut-expressed trypsin, *LsTryp1a*, in these lice. As with many of the labial gland genes, *LsTryp1a* was also found to be differently regulated in lice on pink salmon as compared to lice on Atlantic salmon at 3 dpi (Figure 6K).

### 3.6 Functional knockdown studies

To investigate whether the salmon louse labial gland enzymes have an immune dampening effect on the cutaneous immune response of Atlantic salmon, knockdown of *LsLGA1-5*, *LsLGA7*, *LsLGSP1*, and *LsLGAp1* was induced in nauplii that were allowed to infest Atlantic salmon after molting to copepodids. The cutaneous immune responses underneath knockdown lice were further compared to those of control lice. Moreover, the expression of a selection of labial gland genes was analyzed in control and KD animals, so as to confirm KD of the target labial gland enzyme gene and analyze if KD affected the expression of other labial gland expressed genes as seen for LsLGP1/1L (Øvergård et al., 2022).

**FIGURE 7:**
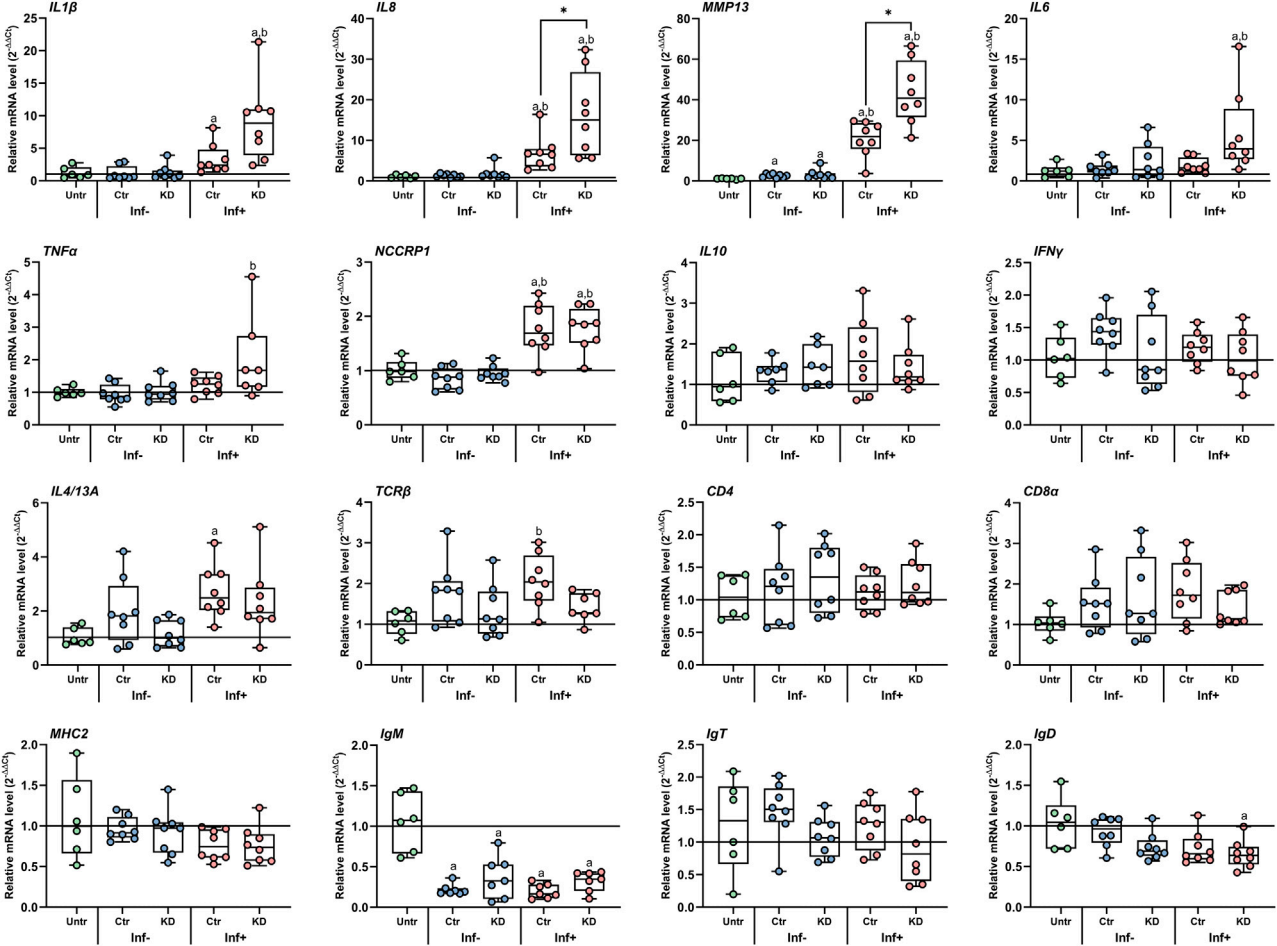
Relative transcript level (2^−ΔΔCT^) of selected Atlantic salmon immune gene transcripts in response to control (Ctr) and *LsLGSP1* KD salmon louse copepodids 3 days after infestation. The expressions in unaffected (Inf-) and affected sites (Inf+) on infested fish were related to the *elongation factor 1 alpha* (*elf1α*) and *tripartite motif* (*trim*) genes (ΔCt) and to non-infested control fish (Untr, ΔΔCt). Each value is plotted in a boxplot showing the median ± interquartile range (N = 8). Statistically significant differences from uninfested fish are denoted with a and between unaffected and infested sites with b. * denotes a significant difference between skin sites infested with Ctr and KD lice. Abbreviations: IL, interleukin; MMP13, matrix metalloproteinase 13; TNFα, tumor necrosis factor alpha; NCCRP1, non-specific cytotoxic cell receptor P1; IFNγ, interferon gamma; TCRβ, T-cell receptor beta; CD, cluster of differentiation; MHC, major histocompatibility complex; and Ig, immunoglobulin.

As previously reported (Øvergård et al., 2018; Dalvin et al., 2021; Øvergård et al., 2022; Øvergård et al., 2023), the immune responses toward copepodids were mainly localized to the site of infestation in all experiments (Figure 7). Hence, comparing the responses at the infestation sites of control and KD lice would be the most appropriate, and indeed, while no differences in the immune responses were seen after *LsLGA* or *LsLGAp1* KD (results not shown), an increased level of the Atlantic salmon immune transcripts *IL1β*, *IL8*, *MMP13*, *IL6*, and *TNFα* was seen underneath *LsLGSP1* KD copepodids 3 dpi (Figure 7). This response was significantly increased for *IL8* and *MMP13*. No regulation was, however, seen for *T-* and *B-*cell markers, *MHC2* and *NCCRP1*, and the cytokines *IL4/13A*, *IFNγ*, and *IL10*. As for the *LsLGAp1* and *LsLGA* genes, *LsLGSP1* showed a highly efficient KD (96% ± 2%). The KD did, however, not affect the number of lice per fish that was similar between the control and KD lice-infested groups in all experiments (for LsLGSP1 KD 36 ± 7.5, control 37 ± 9 lice/fish, Supplementary Figure S1).

## 4 Discussion

Enzymes secreted by parasites are known to catalyze various biological factors at the host–parasite interface and have great potential to degrade and initiate the digestion of host tissue and interfere with host responses. As such, essentially, all known parasites secrete enzymes with varying effects on the host, depending on the infestation strategy of the parasite. The salmon louse is an ectoparasite that inflicts only superficial wounds to its host, at least on healthy fish infested with low lice levels (Jones et al., 1990; Bron et al., 1991; Jonsdottir et al., 1992; Dalvin et al., 2020), and induces a somewhat moderate host immune response in susceptible salmonids (Skugor et al., 2008; Braden et al., 2012; Braden et al., 2015; Dalvin et al., 2020; Dalvin et al., 2021; Øvergård et al., 2023). Thus, enzymes secreted by the salmon louse labial glands are not expected to degrade components in skin tissue that is not ingested as this would probably cause more severe damage than what is generally seen. The labial gland enzymes are, however, more likely to, e.g., actively dampen immune responses, inhibit blood clotting, or interfere with wound healing responses. All the labial gland enzymes identified in the present study contained signal peptides and canonical residues important for enzyme activity, suggesting that they are secreted functional enzymes. The labial gland enzymes are, therefore, likely to be deposited on the host skin during lice feeding, where they are expected to modulate one of the abovementioned functions.

### 4.1 Astacins are suggested to have an anti-microbial function

The astacins are a highly divergent zinc-dependent endopeptidase family, with abundant orthologues genes found in both vertebrates and invertebrates, where many species within the latter group display a similar gene expansion as seen in salmon louse (Skern-Mauritzen et al., 2021; Gomis-Rüth and Stöcker, 2023). Compared to its high number of family members, relatively little is confirmed regarding their function, especially in crustaceans. Therefore, it is difficult to suggest the function of the salmon louse labial gland astacins just based on gene similarity. As the other known labial gland genes have not been shown to have any extra-labial gland expression (Øvergård et al., 2022), it was surprising that *LsLGA1* and -*4* transcripts were localized in a special subtype of teg 1 glands in addition to the labial gland. Teg 1 glands are the most numerous salmon louse gland type, found in all lice body parts, in all lice life stages, and is believed to secrete a mucoid layer with an anti-fouling function covering the lice integument (Bron et al., 2000; Bell, 2001; Øvergård et al., 2016; Harasimczuk et al., 2017). The teg 1 subtypes look similar to the regular and much more numerous teg 1 gland, but do not express *LsAst1* that shows a high expression in the ordinary teg 1 glands (Øvergård et al., 2016). *LsAst1* has been suggested to act as an anti-microbial enzyme, and the same function might, therefore, be advocated for the *LsLGA* genes. It would not only be an advantage to keep the microbial growth on the lice integument to a minimum, but it would also be beneficial to diminish the risk of secondary infections at the inflicted wounds to sustain a long and healthy host–parasite relationship. The high expansion of astacin genes within the salmon louse genome would support such a hypothesis, as the astacins would have to attach to and destroy various types of microorganisms, probably demanding an arsenal of slightly different enzyme specificities. Like the labial gland, the *LsLGA1*- and -*4*-expressing teg 1 subtype secretes its products directly onto the host skin through two relatively large pores at the base of the post-antennary process (Øvergård et al., 2016; Harasimczuk et al., 2017). Thus, a wider distribution of these two LsLGA proteins could be expected in tissues underneath the lice, giving a greater potential for anti-microbial support. Moreover, these large pores are in close proximity to where the sharp tips of the second antennae pierce into the host skin for host attachment (Kabata, 1982; Jonsdottir et al., 1992), and the secrete would, thus, be deposited onto the skin site damaged by both feeding and attachment.

How exactly lice astacins can have an anti-microbial effect is not known, though the answer might lie in their C-terminal ShK domain. At least 19 of the predicted astacin genes within the salmon louse genome encode 1–4 ShK domains (Skern-Mauritzen et al., 2021), including the labial gland astacins identified in the present study. The action of ShK toxins alone has been extensively studied, found to bind the potassium channel, thus possessing both neurotoxic and immunosuppressive properties where each ShK seems to have a selectivity toward relatively few potassium channel subtypes (Honma and Shiomi, 2006; Castaneda and Harvey, 2009). However, the functional information of ShK domains attached to astacins or other enzymes is scarcer. The ShK domains of the pearl oyster (*Pinctada fucata*) Pf-ALMP astacin were found to be necessary for the enzymes to promote cell proliferation (Xiong et al., 2006). The ShK domains of the human matrix metalloprotease 23 (MMP23) were found to block specific potassium channels when expressed alone, while the full-length MMP23 co-localized and trapped the potassium channels within the ER and in that way suppressed their function (Rangaraju et al., 2010). These two examples indicate that ShK domains C-terminally on astacin metallopeptidases could potentially bind to potassium channels, targeting the astacin to its substrate. However, almost all ShK domains on the identified LsLGA proteins did not display a conservation of the functional Lys–Tyr dyad that is known to dock into potassium channels blocking the potassium from entering (Honma and Shiomi, 2006). The ShK domain of MMP23 also lacks the dyad but is believed to interact with the external vestibule of the channel for blocking (Rangaraju et al., 2010). Moreover, almost all the ShK domains of the LsLGA proteins have one or two lysins that seem to protrude from the globular structure, and studies of other K^+^ channel-blocking peptides have shown that lysine in various positions of the peptides can dock into the channel (Stehling et al., 2012). Thus, alternative lysins of ShK domains could potentially dock into the channel vestibule, suggesting that the LsLGA proteins might bind to and enzymatically cleave potassium channels on cells at the host–parasite interphase. Interestingly, potassium channels have been found to enable electrical communication in bacterial biofilms upon nutritional deprivation, as to secure the interior bacterial cells’ access to nutrition (Prindle et al., 2015). Thus, docking into and enzymatically altering the potassium channels of bacteria at the site of feeding could inhibit bacterial colonization. Nevertheless, it should be emphasized that other functions should not be excluded, and even though the present study suggests that the LsLGA proteins do not have an immunosuppressive role, KD effects could be masked if the multiple labial gland astacins have complementary immune dampening function or cleave factors that do not have a direct effect on the analyzed immune gene transcripts. Performing functional studies applying recombinant LsLGA proteins analyzing additional immune functions, neuroimmune signaling, and cell proliferation, in addition to addressing the host microbiome in similar KD studies as presented here, is necessary to conclude on their function.

### 4.2 LsLGSP1 may have a direct or indirect anti-inflammatory function

As opposed to the *LsLGA* and the *LsLGP* genes displaying a relatively high transcript level, both *LsLGSP1* and *LsLGAp1* display relatively low levels of expression. Serine proteases are often very potent proteolytic enzymes that might damage tissues if secreted at high levels. This is of course dependent on the enzyme specificity and mode of activation. Serine proteases are typically produced as inactive zymogens to avoid protein cleavage and tissue damage at the production site and are activated, e.g., upon secretion into the gut lumen (Khan and James, 1998). Activation is a result of enzymatic cleavage at a distinct site, IVGG, that was found to be partly conserved in LsLGSP1. Thus, LsLGSP1 seems to require cleavage to become activated. However, even though the LsLGSP1 cleavage site Ile is preceding an Arg needed for cleavage, it lacks the enteropeptidase consensus sequence (DDDK/R), alike that seen for most other invertebrate trypsins (Muhlia-Almazan et al., 2008). An increase in the number of Asp residues within this consensus sequence is suggested to decrease the tendency of autoactivation, enhancing the action of specific activation by enteropeptidases. Therefore, autoactivation of LsLGSP1 could be expected.

After *LsLGSP1*-KD, an increased transcript level of cytokines typically involved in inflammatory responses was detected underneath the louse, indicating that secreted LsLGSP1 directly or indirectly dampens the inflammatory response at the host skin. If LsLGSP1 acts in tissue degradation, an opposite effect of *LsLGSP1*-KD would be expected, as it would lead to a decrease in tissue damage and inflammation. Interestingly, LsLGSP1 shows similarity to invertebrate serine protease stubble and svh-1 and to the vertebrate type II transmembrane serine proteases such as hepsin, prostasin, and matriptase. Svh-1 is necessary for *C. elegans* larval growth, evidently cleaving the extracellular matrix (ECM) protein fibulin that, in Svh-1 mutants, accumulates around the pharynx interfering with normal pharyngeal pumping during feeding (Hisamoto et al., 2014). The substrate for the *Drosophila* serine proteinase stubble is not known, although it is required for epithelial morphogenesis during development of bristles, legs, and wings where it is believed to modify the ECM, permitting changes to the epithelial cell shape (Hammonds and Fristrom, 2006). Hepsin, prostasin, and matriptase do also have their function in epithelial or epithelioid tissues, matriptase, and prostasin in corneocyte differentiation, likely by a proteolytic activation of epithelial sodium channels (Miller and List, 2013), while hepsin regulates hepatocyte morphology and growth cleaving a pro-hepatocyte growth factor (Li et al., 2021). As lice larva, particularly the chalimus stage, are restricted to a small area of the host skin where they feed mainly on the epithelial surface (Dalvin et al., 2020; Heggland et al., 2020), it could be an advantage to increase the level of epithelial cell proliferation at the site of attachment. This would sustain access to nutrients and aid in wound healing, thus indirectly acting as immune dampening, and in fact, epithelial cell proliferation seems to be a response to copepodid attachment (Øvergård et al., 2023), though it is not known if this is mainly a wound healing response or if the lice can enhance such responses. In the resistant coho salmon, epithelial cell proliferation develops into a hyperplastic reaction that covers and kills the lice (Johnson and Albright, 1992). Infesting coho salmon with *LsLGSP1*-KD lice could, thus, give some answers. Nevertheless, further research on LsLGSP1 should investigate its potential as an activator of salmon epithelial cell growth factors, in addition to its ability to cleave pro-inflammatory immune components.

### 4.3 LsLGAp1 is unlikely to inhibit blood clotting

Skin wounding will release adenosine, AMP, ADP, and ATP, into the extracellular milieu that will stimulate various responses at the wound upon binding to the purinergic receptor (Burnstock et al., 2012). Thus, secretion of apyrases to control such responses is expected to be beneficial for a parasite inflicting skin wounds on its host. As each nucleotide signals through different receptors, the epithelial and endothelial response to the different nucleotide varies, and hence, the function of LsLGAp1 depends on its nucleotide specificity. When endothelial cells are damaged, they release ADP that can bind to purinergic receptors on platelets inducing platelet aggregation and plug formation. Many blood-sucking arthropods, therefore, secrete apyrases at the feeding site that can hydrolyze ADP to inhibit blood clotting (Smith and Kirley, 2006). The expression pattern of LsLGAp1 indicates, however, that it does not have an anti-clotting function, as a more similar expression pattern to the anti-clotting protein LsLGP4 would be expected. LsLGP4 is mainly expressed in pre-adult and adult lice (Øvergård et al., 2022), where hematophagous feeding is initiated (Heggland et al., 2020), while LsLGAp1 seems to have a more constitutive expression throughout all the parasitic life stages of the lice, also when the lice mainly feed on the epidermal surface. Moreover, LsLGAp1 shows a higher similarity to the 5′-nucleotidase/CD73 enzyme family of apyrases, and even though both *A. aegypti* and, maybe, the soft tick *Ornithodoros savignyi* use members of this apyrase family to inhibit platelet aggregation (Champagne et al., 1995; Stutzer et al., 2009), AMP is the preferred substrate for CD73 (Heuts et al., 2012). In mammals, CD73 acts together with the apyrase CD39, by converting ATP/ADP to AMP (CD39) and AMP to adenosine (CD73) (Antonioli et al., 2013). ATP can act as a danger-associated molecular pattern (DAMP) when released from injured cells or immune cells, activating T helper cell proliferation, cytokine release, and inflammation. Therefore, catalyzing ATP to adenosine induces a shift from an ATP-driven pro-inflammatory environment toward an anti-inflammatory environment, as adenosine has immune-suppressive effects; e.g., in human skin, adenosine inhibits neutrophil influx and the release of reactive oxygen species (ROS) and decreases skin edema (Burnstock et al., 2012). Thus, based on LsLGAp1 ubiquitous expression in all parasitic salmon louse life stages, it is tempting to suggest such an immune suppressive function for LsLGAp1. However, *LsLGAp1*-KD lice did not induce an increased local immune response at the lice attachment site compared to control lice. Thus, the net effect of LsLGAp1 on the host immune response is not great, maybe correlating to its low expression level. Interestingly, both keratinocytes and sensory nerve cells are responsive to ATP released upon the mechanical stimulation of human epidermal keratinocytes, and ATP is known to induce pain responses (Ulmann et al., 2007; Burnstock et al., 2012). Moreover, both ATP and adenosine are found to be involved in various parts of skin wound healing (Burnstock et al., 2012). Thus, the main effect of LsLGAp1 could be many. Further studies are, therefore, warranted to elucidate the substrate specificity of LsLGAp1, as this will give further hints to its function at the lice feeding site.

### 4.4 Labial gland proteins might be important for successful host establishment

The parasitic copepodid stage is the most vulnerable phase during the salmon louse parasitic phase, as its grip on the host is not as firm as in the following stages that either attach via a frontal filament (chalimus) or a combination of vacuum underneath a suction cup-shaped cephalothorax and the second antenna (pre-adult and adult) (Kabata, 1982). The copepodids attach by burrowing their second antennae into the host skin causing epidermal disruption (Jones et al., 1990), and without another firm attachment point, they are more vulnerable for detachment due to anti-lice behavior and immune responses. Hence, it is important to dampen host responses immediately after attachment, and it could, therefore, be vital for the copepodids to produce the labial gland secretions prior to encountering a host. In line with this, the induction of most labial gland transcripts was not found to be dependent on host attachment, as indicated by the initial ontogenetic analysis of both the labial gland enzymes and the previously characterized LsLGPs (Øvergård et al., 2022; Figure 3). Instead, the production of labial gland secretions was initiated already in young planktonic copepodids around 1–2 dpm (9°C), further increasing until it leveled out at around 4 dpm, and more interestingly, the expression of almost all genes displayed no subsequent elevation if lice were allowed to infest fish. The exact age of the planktonic copepodids analyzed in the initial ontogenetic analysis is unknown, but given the low transcript level of labial gland genes, it was probably taken prior to the induction of labial gland protein expression. Interestingly, copepodids at 10°C have been shown to require 1–2 days of maturation after molting before they become infective at 10°C (Skern-Mauritzen et al., 2020), and the activation of labial gland secrete production, thus, seems to be part of this maturation.

During this pre-infective copepodid maturation time, LsLGP1/1L seems to be a key activator regulating the transcript level of most proteins included in the labial gland secretions at the copepodid stage (Øvergård et al., 2022; Figure 1). *LsLGP1/1L* KD did, however, not affect the transcript level of *LsLGA2*, *8*, and *LsLGP3. LsLGP3* is mainly expressed in the labial gland’s reservoir part (Øvergård et al., 2022); thus, LsLGP1/1L might not regulate gene expression in this labial gland compartment. Moreover, the lack of *LsLGP1/1L*-KD-induced regulation of *LsLGA2* and -*8* might be explained by the fact that both transcripts seem to decline in parasitic copepodids, and *LsLGA2* was found to decrease as a response to host attachment. As LsLGA2 is highly similar to LsLGA1, -3, and -4, whose transcript levels are increasing after host attachment, the biological implication of a declined *LsLGA2* level might be minimal. Nevertheless, it shows that host attachment to some degree regulates the labial gland secrete composition, also as the induction of *LsLGP3* and *LsLGA7* was found to be host dependent. LsLGP3 is suggested to dampen cellular responses (Øvergård et al., 2022), and as the migration of immune cells to the site of lice attachment is not seen immediately after infestation (Dalvin et al., 2020), it is probably not critical to secrete LsLGP3 at the very early stage of the establishing phase. Suppressing cellular responses could, thus, be a common trait for these two proteins, and further studies should more thoroughly investigate the immunomodulatory role of LsLGA7. LsLGA7 is the only astacin within the salmon louse genome having as many as four ShK domains, and different to the other LsLGA proteins, these domains are connected with relatively long linker sequences. Additionally, the LsLGA7 ShK domains have the highest degree of Lys–Tyr dyad conservation and, thus, have a higher probability of being able to dock into potassium channels. T-cells are dependent on potassium channels for Ca^2+^-influx and activation, and synthetic ShK domains have, therefore, been designed to bind their potassium channels with an immune-dampening effect (Selvakumar et al., 2022). Thus, it is tempting to suggest another role for LsLGA7 than that suggested for the other LsLGA proteins. However, *LsLGA7* demonstrates a relatively low expression level in the mobile stages in contrast to *LsLGP3* that shows a steady increase throughout the parasitic life stages, correlating with an increase in immune cell influx (Dalvin et al., 2020; Øvergård et al., 2022). Thus, other LsLGA7 functions than immune cell modulation should be considered, focusing on effects mainly inflicted by the salmon louse larva.

A relatively high transcript level for many of the labial gland genes is, in fact, seen in the salmon louse larval stages (Øvergård et al., 2022; Figure 3), further emphasizing the need to dampen responses during this first establishing phase. Early in the copepodid stage, the attachment and feeding activity causes minor disruption of the epidermis (Jones et al., 1990), with only minor penetration of the basement membrane seen (Bron et al., 1991). In the chalimus stage, skin erosions become more severe as the chalimii grow, and it has a frontal filament that in Atlantic salmon attracts only a limited number of immune cells even though it penetrates the epidermis (Jones et al., 1990; Johnson and Albright, 1992). A stronger dampening of the immune response during this phase is, thus, likely needed for lice settlement and also to avoid frontal filament rejection. Nevertheless, the expression of labial gland enzymes during the mobile stages also seems to be important, as all enzyme families are represented at these time points; *LsLGA4* have a steady increase and *LsLGSP1* and *LsLGAp1* with a more constitutive expression. Particularly in the adult stage when an increase in both size and feeding activity is seen, labial gland expression seems to increase (Øvergård et al., 2022; Figure 3). Interestingly, both the rainbow trout (*Oncorhynchus mykiss*) and Atlantic salmon immune response toward adult salmon louse seem to be more suppressed compared to earlier stages (Dalvin et al., 2020; Øvergård et al., 2023), correlating with this increase in labial gland protein expression (Øvergård et al., 2022; Figure 3). Moreover, as previously seen for the LsLGPs (Øvergård et al., 2022), none of the labial gland enzymes were found to be regulated by starvation. This further emphasizes the importance of the labial gland secrete, as copepodids have apparently not evolved any starvation-induced energy-saving mechanisms with respect to the production of these proteins, as typically seen for genes expressed in the gut (Heggland et al., 2019a; Heggland et al., 2019b; Figure 5). Instead, labial gland secrete production appears to be sustained, allowing immediate immune dampening at the site of attachment when re-establishing on a potentially new host.

### 4.6 Labial gland gene expression is sustained in lice infesting pink salmon

The salmon louse shows variable survival on different salmonids, and particularly, pink and coho salmon are far more resistant to the lice than other salmonids (Jones, 2011). It is, therefore, of interest to analyze whether labial gland gene transcripts are produced in lice that infest resistant species, as a first step toward investigating the regulation and function of labial gland factors secreted onto resistant species. As most labial gland transcripts are induced already prior to host attachment, it was not surprising to find that most genes were also expressed in lice infesting pink salmon. Many transcripts showed, however, a slightly higher transcript level in lice on pink compared to those on Atlantic salmon. This might be a compensatory effect to a hostile environment, or maybe the lice that manage to establish on pink salmon have a somewhat higher transcript level of labial gland proteins compared to the average level seen on lice that establish on Atlantic salmon. A systemic effect could, however, also be causing this higher transcript level if the lice on pink salmon grow slightly less than the lice on Atlantic salmon; thus, the gland size compared to the rest of the body ratio would be higher in lice on pink salmon at 3 dpi, thus also the transcript level of labial gland proteins. Interestingly, the digestive enzyme LsTryp1 was also found to be expressed at a slightly higher level in lice infesting pink salmon, indicating that lice copepodids feed on the epidermis in both species. On the other hand, *LsLGA7* does not seem to be induced upon pink salmon attachment, while *LsLGP3* was expressed at the same level, even though expression of both genes is induced upon host attachment to Atlantic salmon. Thus, the signal that activates the gene expression of *LsLGP3* and *LsLGA7* is different and further points in the direction of different functions. Nevertheless, the present study shows that the level of most labial gland transcripts is sustained when lice are infesting pink salmon, and labial gland gene expression has also been seen in lice infesting coho salmon (Braden et al., 2023). Further analysis should, thus, evolve around the function of the labial gland proteins. If key labial gland proteins and enzymes are non-functional when meeting the skin and immune factors of resistant salmonids, this can explain the apparent lack of local immune modulation underneath lice infesting these salmon species (Braden et al., 2012; Braden et al., 2023). However, other resistance mechanisms may very well be in play.

### 4.7 Concluding remarks

In conclusion, the present study successfully identified and characterized enzymes expressed in salmon louse exocrine glands that are expected to deposit the secrete directly onto the lice feeding site at the host skin. These enzymes are, therefore, likely to be important for modulating host responses. Based on the gene expression and sequence analysis along with functional KD studies, an indirect or direct immune-modulatory role of all enzyme families could be suggested. As no decrease in inflammation was seen underneath KD animals, the proteases do not seem to act in tissue degradation, and instead, an increase in pro-inflammatory genes supports an anti-inflammatory role for LsLGSP1. We also suggest that the LsLGA proteins have anti-bacterial properties, which could prevent secondary infections at the lice feeding site, further reducing inflammation at the site of attachment. LsLGA7 seems, however, to differ from the other LsLGA proteins, both when it comes to gene regulation and the conservation of key residues important for potassium channel docking, and might, thus, be more directly involved in immune modulation. Moreover, a constitutive expression of LsLGAp1 in all parasitic lice stages at least suggests that it is not important to inhibit platelet aggregation, rather its role is at the epidermal surface. All in all, 15 labial gland expressed genes are now identified, where some of these are likely to have overlapping functions. Thus, KD studies often result in only indicative phenotypic alterations, and studying the action of recombinant labial gland proteins would be a natural next step toward understanding their function. Such knowledge may aid in developing enhanced immune-based anti-lice treatments, especially since the importance of the labial gland secrete is here emphasized by the finding that secrete production is initiated already before the copepodid infests its host and is expressed in lice infesting the resistant pink salmon.

## Data Availability

The data presented in the study are deposited in the https://www.ncbi.nlm.nih.gov/genbank/ repository, accession number: OR504246, OR504244, OR504245, OR504243, OR504248, OR504249,OR504247, OR504250, OR504241, OR504242, and deposited in the https://www.ncbi.nlm.nih.gov/ repository, with accession number PRJNA1008109, and PRJNA1008109.
