# Supplementary material for: Salmon louse labial gland enzymes: implications for host settlement and immune modulation
Source: Front Genet. 2024 Jan 17;14:1303898. doi: 10.3389/fgene.2023.1303898 (PMC10828956; doi:10.3389/fgene.2023.1303898)
Supplement: Supplementary file 1 [file DataSheet2.PDF]

Supplementary file 2

Astacins

The astacin genes were after RACE found to be between 1066-1661 nt long, with ORFs encoding from 310 to 473 amino acids. The metallopeptidase domains were quite similar in size, and all were found to contain the conserved zinc-binding and catalytic site consensus sequence (HExxHxxGxxH) immediately followed by a family-specific glutamate as part of the consensus motif ExxRxDRD (Fig. 1). The last negatively charged aspartic acid within this motif was, however, found to be substituted with the similar but uncharged aspargine in LsLGA8. Moreover, some substitutions were also seen in the downstream SIMHY-motif that in other astacins encompasses a “Met-turn” methionine and a zinc-binding tyrosine (Gomis-Rüth and Stöcker 2023). It was fully conserved in all LsLGAs but LsLGA5 and 7, where isoleucine was found to be substituted by the similar amino acid residues leucin or valine.

After the astacine domains, all genes encode a short linker sequence rich in threonine and proline followed by either 1, 2 or 4 *Stichodactyla helianthus* potassium channel toxin (ShK) domains. The ShK domain is 35-37 amino acid residues long with six cysteines scattered along the domain in a distinct pattern forming three disulfide bridges stabilizing a globular tertiary structure (Honma and Shiomi 2006). All LsLGA ShK domains show conservation of all six cysteines (Fig 2), predicted to form the canonical disulphide bridges between cysteine 1 and 6, 2 and 4, and 3 and 5. Only the second ShK domain of LsLGA7 displayed a conservation of the functional Lys-Tyr dyad, with the first and the third ShK domain of LsLGA7 and the second ShK domain of LsLGA6 having only the lysine. However, other lysine residues were predicted to protrude from the globular tertiary structure in one or more ShK domains of all LsLGAs besides LsLGA2 and 4 (Fig 3).

As the combination of astacins and ShK domains is restricted to invertebrate species, BlastP searches (NCBI) was done with the astacin domains only. All LsLGA astacin domains shows the highest similarity to one or more *Caligus rogercresseyi* astacins, besides LsLGA8 that were found to have the highest similarity to astacin sequences from the primitive ray-finned fishes *Polyodon spathula* and *Acipenser ruthenus* annotated as high choriolytic enzyme 1-like. The other LsLGAs also showed some similarities with astacins from fish, though LsLGA4-7 were more alike various insect astacins particularly from flies and mosquitos with various annotations. Limiting the BLAST search to invertebrate species with more functional data available, the LsLGAs were found to have the highest resemblance to *Ixodes scapularis*

Nas-14, *Caenorhabditis elegans* Nas-15 and *Aedes aegypti* Nas-7 (LsLGA1 and 7), 4 (LsLGA2 and 6) or 14 (LsLGA3, and 8) in addition to a seminal metalloproteinase 1 (LsLGA4) and a gene only annotated as astacin (LsLGA5). We also looked into the similarity between the LsLGAs and the LsAst1 found to be expressed in tegmental type 1 glands (Øvergård, Hamre et al. 2016). LsAG1-4 have a 78-83 % sequence similarity to each other, while LsLG5-8 displayed a sequence similarity of around 34-53 % to each other and to LsAG1-4 (Table 1). Interestingly, LsLGA6 was more similar to LsAst1 than to the other LsLGAs, with a similarity of 67 %.

```
LsLGA8     ------RNIV---DSRWPRGKVPYKFTSNYSR-KEINLIRRAMKDIQSRSCVKFVKARPKH-THYVDIGSDQKGCHASLGFN
LsLGA6     -------NFIGGRSNRWPNGIVPYTISSDFSS-SQRQTIMDAFKHISDRTCVTYVE-RTNE-ANYLDIFTGGDGCFANIGYN
LsAst1     ------RNFIGGRSNRWPNGIVPYTISSNFSS-SQRQTIMDSIKHISDRTCVKFVQ-RTNQ-ANYVDIFTGASGCNANLGYN
LsLGA5     ------RNYVSDERKHWPNKEVPYTISNSYPP-KYNKLLRKAMKAIESMSCVKWIP-RTNQ-RNYVYISPKQSGCFANLGYD
LsLGA7     -------NLL--GGKVWPNNTVPYILAGGFSK-DDLSLIQSAMDGIEKKTCVRWVP-RNGE-KSYVYIKNDESGCFAVLGYN
LsLGA2     -------NFV--GGEKWPNNEVPYEISFGYNR-YEVSVIKSAMKEIESKTCVKWVP-RSGQ-KNYVTINPREKGCFAVMGYN
LsLGA4     -------NFI--AGEKWPNNEVPYEISSEYSS-SDVDVIKSAMKEIGNKTCVKWVP-RSGE-KNYVLINPQQRGCSAVLGYN
LsLGA1     -------NFI--AGAKWPNNEVPYEISSGYSS-SDLDVIKSAMREIESKTCVKWVP-RSGQ-KSFVLINPREDGCFAVLGYN
LsLGA3     -------NLI--AGENWPNNEVPYVISSEYSS-SDVDVIKSAMKEIESKTCVKWVP-RSGQ-KSYVTINPREEGCFAVLGYN
IsNas14L   --------GVKDETLLWPEGKVFYKIDKGFDSDSDRKVILDAMELLQSKTCVKFIE-RTDE-ENYVLIAPRPGRCASFVGMQ
CeNas15    -------NAIKNRLQLWPEGRIPYTISSQYSS-YSRSLIAASMQEYASHTCIRWVP-KEAADVNYVHIYP-DRGCYSMVGKM
SsHE1.2L   NAMICFSAGCFWKKGSNGLVEVPYTVSSSFSS-SDKQSIENALRAFPSKTCIRFVP-RQNQ-VDFISYEP-RDGCWSSLGRV
AaNas7     -------NAVKFKSRKWAKAVVPYKISDSFGY-SDVQRIQMAFKMFHQKTCVRFVP-RASQ-RDFIAIEKSSSGCWSTVGRA
                                : * .   :      . :  ::    . :*: ::  :      ::        * : :*

LsLGA8     KDIGRHYVNLERP----GCMFLSGIQHELLHILGFDHEQNRPDRNSYVRINWQYLNQDSFKSFFRSVEKSTAPLPQKCDPSR
LsLGA6     XRRGRSQLHLQSN----GCVVLGIITHELLHILGFSHEQTRPDRDQYVKINWENLKIGTHSNFWRALGENEPAAIPYCGSVG
LsAst1     SGRARSVLHLQSD----GCVHLGIIVHELLHILGFAHEQTRPDRDQYVTISWENIRSGTYSNFWRALGENEAVTIPYCGIVG
LsLGA5     VRRGKHTLNLQMPHGRSTCMVLGIAMHEMLHILGVGHEQCRPDRDSYVKVHWRNMENDKYNNYFKSIGPKTQTRMPVCDPKK
LsLGA7     EYRGKHVLNLQRSNGFSTCMIFGIAQHEMLHILGYGHEQTRPDRDSYVRIHWDMIQRDAISNYFKSIYDNTTIVPPQCRPRS
LsLGA2     RNRGVHNLNLQRNNGYTTCMSMRIAAHEMLHILGFAHEQTRPDRDQFVRIYWSRIKRSSISNYFRAIDINSRERPPVCDPMS
LsLGA4     RNRGVHNLNLQRNDGSTTCMIMGIAAHEMLHILGFAHEQTRPDRDQFVQIFWSRIKRNYISNYFRAIDINATDIPPVCDSTS
LsLGA1     PNRGMHVLNLQRSNGRSTCMIMGIAAHEMLHILGFAHEQTRPDRDQFVQIYWSRIKRDSISNYFRAIDINARERPPVCDPRS
LsLGA3     RNRGVHNLNLQRTNGGRTCMIMGIAAHEMLHILGFAHEQTRPDRDQFVHIYWSRIQRDSISNYFRAIDINARERPPVCDPRS
IsNas14L   --GEEQALALDLY----RCPNVGQSVHELMHAIGFFHEHSRPDRDSYISIQWNNIEEDAKQSFELKTE--------------
CeNas15    --GGKQSLSLGS-----GCIQKGIILHELMHAVGFFHEQSRTDRDDHITIMWNNIQAGMQGQFEKYGH--------------
SsHE1.2L   --GGQQTVSLQMD----GCVYFGVIQHETLHALGFQHEQTRSDRDQYVEINWSNIDSNNAYNF---DR--------------
AaNas7     --GGRQVVNLQSS----CLRQIGTVLHELMHALGFLHEHTRHDRDKFVDINYLNVRFDAIGNFWKETK--------------
                  : *                ** :* :*  **: * **:..: : :  :  .   .:

LsLGA8     -GYTFDDCYSGYFTDTLGLPYDYMSIMHYAKDSFTKYPGYNVMEPLQQ--GGENM---GKSRKMTDLDHDKLYKAYGCK 222
LsLGA6     -VDQYDNCYAGFRASTFGLNYDYGSIMHYGLRYFSTN-GRNTMTLKKST--TAQI---PNRRGMSSLDVQKTKLAYECQ 222
LsAst1     -IDQYDNCNAGFRTSTFGMAYDYGSIMHYGLTYFSTN-GQNTITLKKST--TARI---PNRSGMSDLDVQKTKAAYECQ 223
LsLGA5     -RMDFDRCWSGYRTNTFGFAYDYQSLMHYGLNDFTQT-GYPTMSTKKPVPSGIKI---GQRIGMTRLDVSKLNAAYKCQ 229
LsLGA7     TATTFDDCYSGFTTDTFGYPYEYGSVMHYGLDDFQTS-DKNTMDVLRPVPFGIRI---GQRIGMTELDALKVKAKYNCD 227
LsLGA2     TQASFDNCYSGFKTRTFGLDYDYGSIMHYGLKDFTTT-TQDTMRVLKPVPPGIVI---GKAEAMTKLDALKVQARYDCN 227
LsLGA4     TQASFDNCYSGYKARTFGLSYDYGSIMHYGLNYFTST-GQDTMGILKTVPVGAVI---GNRNGMTKLDALKVQARYDCN 227
LsLGA1     TQTSFDNCYSGFKTRTFGLEYDYGSIMHYGLDDFTTT-GQDTMRVLRPVPTGIVI---GNRKGMTNLDALKVKMRYDCN 227
LsLGA3     TQTSFDNCYSGFKTRTFGLRYDYGSIMHYGLNYFTST-GQDTMGILKPVPVGAVI---GNRKGMTKLDALKVQARYDCN 227
IsNas14L   -----------LIADTLGQPYDYESVMHYPQDAFSKSPPTPTLIPKSPDIDPATLGKGYLENFLTDTDVIKVNLLYGCA 202
CeNas15    -----------GTIQSLGTGYDYGSIMHYGTKAFSRNGQ-PTMIPKKN-G--ATI---GQRNGFSKVDKFKINTLYGCP 194
SsHE1.2L   -----------SNTNNLNTPYDYSSVMHYGKTAFSINGM-DTITPIPNPN--VSI---GQRQGLSTTDILRINRLYSC- 198
AaNas7     -----------DRTSTLGTGYDLGSVLHYSRKAFSWNGF-NTIDPKVKFS--GKI---GQRNGFSNSDIKRIKAMYCN- 195
                          .:.  *:  *::**    *       .:           :        ::  *
```

Figure 1. The astacin domains of *Lepeophtheirus salmonis* labial gland astacins (LsLGA) 1-8 aligned with the astacin domain of *Lepeophtheirus salmonis* astacin 1 (LsAst1) expressed in tegumental type 1 glands (EMLSAT00000007463), *Ixodes scapularis* zinc metalloproteinase nas-14-like (IsNAS14L, XM_040213241), *Caenorhabditis elegans* zinc metalloproteinase nas-15 (CeNas15, NM_075753), *Salmo salar* hatching enzyme 1.2-like (SsHE1.2L, XM_045707985) and *Aedes aegypti* zinc metalloproteinase nas-7 (AaNas7, XM_021842178). The zinc-binding site consensus sequence (HExxHxxGxxH) marked in black was conserved.

The family-specific glutamate as part of the consensus motif ExxRxDRD is highly conserved (marked in green), except from a substitution of the last negatively charged aspartic acid with the similar but uncharged asparagine in LsLGA8. The downstream SIMHY-motif marked gray also had one substation in LsLGA5 and LsLGA7.

Table 1. Sequence identity between the *Lepeophtheirus salmonis* labial gland astacins (LsLGA) and *Lepeophtheirus salmonis* astacin 1 (LsAst1) expressed in tegumental type 1 glands, calculated in Clustal omega. The intensity of green increases with higher similarity.

	**LsLGA8**	**LsLGA6**	**LsAst1**	**LsLGA5**	**LsLGA7**	**LsLGA2**	**LsLGA4**	**LsLGA1**	**LsLGA3**
**LsLGA8**	100	34.31	34.31	37.54	39.07	38.74	36.75	37.42	38.08
**LsLGA6**	34.31	100	67.42	38.49	37.5	38.99	43.45	41.37	40.48
**LsAst1**	34.31	67.42	100	36.28	37.5	39.29	41.67	40.18	40.48
**LsLGA5**	37.54	38.49	36.28	100	45.12	45.71	45.08	46.35	46.67
**LsLGA7**	39.07	37.5	37.5	45.12	100	49.42	47.69	53.76	51.73
**LsLGA2**	38.74	38.99	39.29	45.71	49.42	100	78.45	79.89	82.18
**LsLGA4**	36.75	43.45	41.67	45.08	47.69	78.45	100	79.89	82.76
**LsLGA1**	37.42	41.37	40.18	46.35	53.76	79.89	79.89	100	83.62
**LsLGA3**	38.08	40.48	40.48	46.67	51.73	82.18	82.76	83.62	100

```
HsMMP3      ----------------------------CLDRL--FVCASWARRGFCDARRRLMKRLCPSSCDFCYE       37
ShK         --------------------------RSCIDTIPKSRCTA--FQ--CKHSMKYRLSFCRKTCGTC--       35
LsLGA5_1    ------PHPPHRKTTRK-VYTYGPTSPRCWDEYP-EWCEG--HNGLCYIGYFYMKENCALTCEFCDN       57
LsLGA2_1    -------------EDPG-TKTTRKPSVECKDIY--IQCPS--LTSYCES-RPEIKRGCKVSCGEC--       46
LsLGA3_1    -------------EGPE-AKTTRKPSVECKDKY--IQCPS--LTSYCQS-NPSVKDGCKVSCGEC--       46
LsLGA1_1    -------------EDPE-KKTTRKPSVECKDIY--RECPL--YKNQCKT-NQFIKDGCKVSCGEC--       46
LsLGA4_1    -------------EDPE-TKTTRKPSVECKDLY--IQCPL--YKKYCES-HQSIKDGCKVSCGEC--       46
LsLGA6_1    --------DANTTPAPTTTTPASPTSKPCDDTF--KYCSD--YKHLCGK-NEYMQNHCKKTCFNC--       52
LsLGA2_2    --------------------------TECLNMY--TNCPE--LGHLCGE-NDEITNSCKLTCGLC--       34
LsLGA1_2    --------------------------TECYDMY--RNCPD--MKHLCGE-LDAITKGCKLTCRLC--       34
LsLGA3_2    --------------------------TECYDDY--TNCPD--LGHLCGE-NDGVTEGCKLTCRLC--       34
LsLGA4_2    --------------------------TECYDKY--SNCPV--LGYLCGE-DDTITKDCKLTCRLC--       34
LsLGA6_2    ---------------------------VCENIYGDDGCSQ--LTGYCE--LKIIQKYCQNTCKKC--       34
LsLGA7_4    -------------PGQG-QTTTKKPDTNCVDSS--SNCEG--LKDFCY--HSYLKENCKATCGIC--       45
LsLGA8_1    ----------------E-STTTTTSVAPCEDIY--TNCNV--LTQWCR--DQDKRDNCRKTCRYCQ-       43
LsLGA7_3    ------PEDINTTTTTT-FTTANTTTSDCFDKF--DTCGE--YLSYCS--DKMIADNCPRSCQLC--       52
LsLGA7_1    QLSTLSTTSPT-NASTS-PTNATTTVSVCEDKW--IYCQS--MPEWCS--NKDTSKNCQKTCKLC--       57
LsLGA7_2    ------PEDIN-TTTTT-FTTPNTTTSVCEDKW--IYCDS--MREWCP--FKYTSQNCRKTCKLC--       51
                                        * :      *      *          *  :*  *
```

Figure 2. The ShK domain of human matrix metalloproteinase 23 (HsMMP3, AJ005256) and the *Stichodactyla helianthus* potassium channel toxin (ShK, AB595206) aligned with the ShK domains of the LsLGAs. The conserved cysteines forming disulfide bridges and stabilizing the globular terterary structure are marked in black. The Lys-Tyr dyad that can block potassium channels is marked in green, which was only fully or partly conserved in ShK-domains in LsLGA5 and LsLGA7. Other potential potassium channel docking lysine residues are marked in blue, predicted to protrude from the globular structure in AlphaFold2. Threonine and proline residues in the linker sequence is marked with lighter and darker grey, respectively.

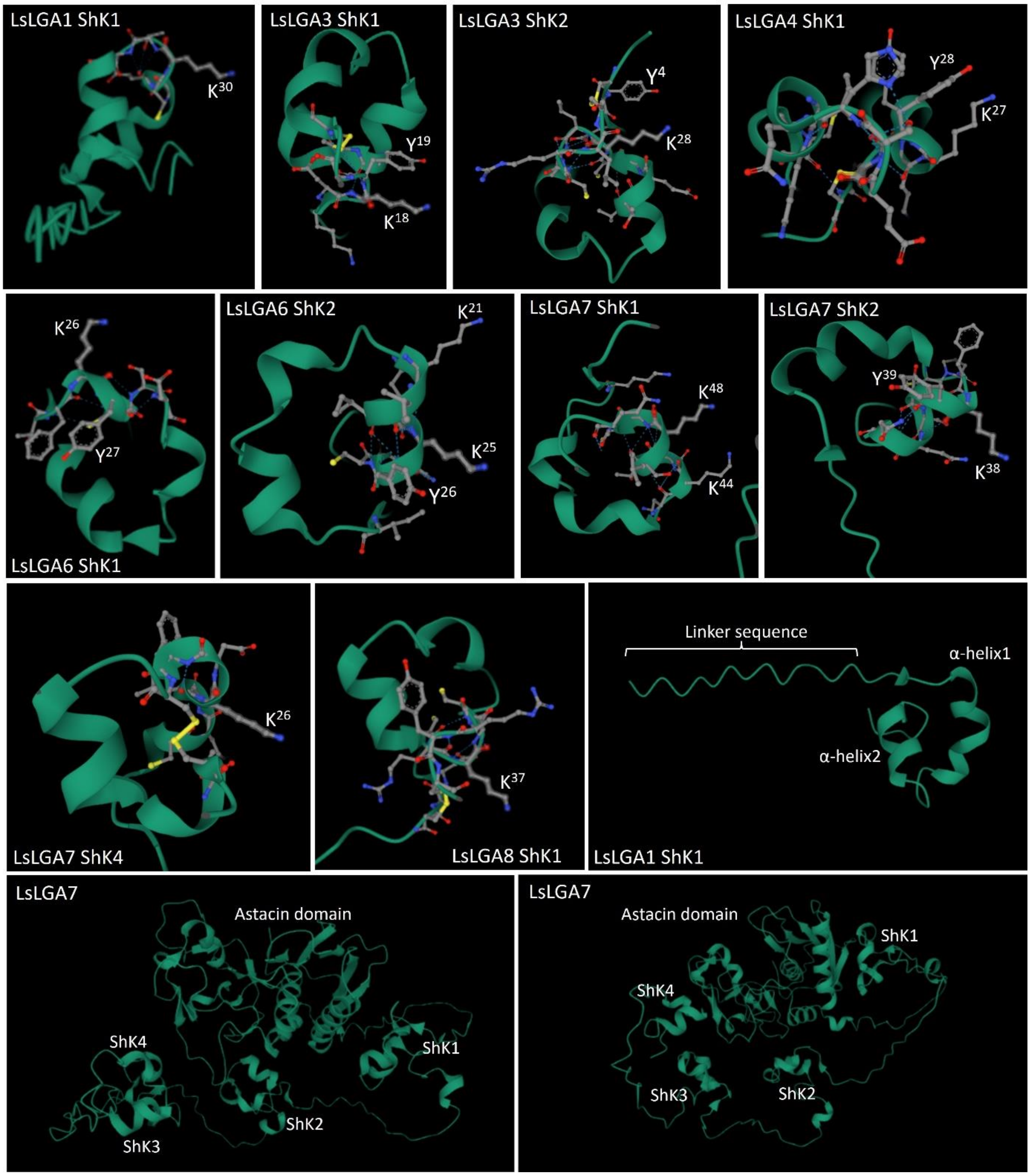

Figure 3. AlphaFold2 prediction of protein folding, showing the ShK domains where lysine residues were found to protrude from the globular structure of the domain, hence maybe taking part in potassium channel docking. If a nearby tyrosine residue is also present, this is marked. The numbering of the residues is related to the numbers in the alignment (Figure 2). An overview of a typical LsLGA ShK domain is exemplified with the first ShK of LsLGA1, where a linker region is seen as an elongated region prior to two alpha helixes making up the ShK domain. The astacin domain of LsLGA7 is also shown together with its four ShK domains.

Serine protease

LsLGST1 was after RACE found to be shorter at the 5'-end as compared to the 1351 nucleotide (nt) long sequence predicted from the salmon louse genome (EMLSAT00000010949), leaving only 1262 nucleotides including the polyA tail. A signal peptide was not predicted in the original sequence; however, this was predicted in the protein sequence encoded from the new truncated ORF. After the signal peptide, putative trypsin and chymotrypsin domain was found. Both are serine proteases that have an active site serine, that together with a histidine and an aspartic acid, forms a catalytic triad that catalyses the hydrolysis of amid bonds (Muhlia-Almazan, Sanchez-Paz et al. 2008). The catalytic triad residues, His, Asp and Ser, were found to be conserved in LsLGST1 (Fig. 4). Trypsin-like serine proteases have a negatively charged aspartic acid in their substrate binding pocket and can therefore specifically cleave the amide bonds after the positively charged amino acids arginine and lysine. The specificity pocket of LsLGSP1 was accordingly found to consist of Asp, Gly, and Gly, indicating it is a trypsin (Perona and Craik 1995). Moreover, an activation cleavage site Ile (IVGG) was identified within a partly conserved consensus sequence I^{60}IGG, where the cleavage site Ile is preceding an Arg, but lacks the enteropeptidase consensus sequence, DDDK/R. No potential sites for GPI-anchoring were identified, whereas one potential N-linked glycosylation site were identified with a weak probability.

BlastP searches (NCBI) revealed that LsLGST1 showed the highest resemblance to an unannotated *Caligus rogercresseiy* chymotrypsin and a prostasin-like gene in *Eurytemora affinis*, followed by various insect and decapod trypsin-like genes annotated as trypsin 1-like, trypsin 7-like, transmembrane protease serine 9-like, venom protease, proclotting enzyme-like, serine protease filzig-like and serine protease stubble-like. Looking specifically into invertebrate species with more functional data available, LsLGST1 was found to have the highest resemblance to *Ixodes scapularis* serine proteinase stubble, *Caenorhabditis elegans* serine protease svh-1 and *Aedes aegypti* venom protease. Limiting the blast search to vertebrate species, LsLGST1 showed the highest resemblance to type II transmembrane serine proteases, particularly hepsin, prostasin and matriptase in various fish species.

```
LsLGST1         ------------------LTSCGDVKRQMNINRIIGGRFANENEIPWSARIVICRSRNECYVCGGTLINHRYVLTAAH
Ce_Svh-1        --------------------------AKSRIARVVGGFETVPGAFPWTAALRNKA--TKAHHCGASILDKTHLITAAH
Is_Stubble      ----------PSKAGMVKDALCG--KTYLRNSKVVGGENAKFGQQPWQAAVVKRSFLSQKISCGGALVHERWVLTAAH
Ss_prostasin    LCLVNLLVAFLSKGSHSQLDVCG--T--PLNTRIVGGQDALAGSWPWQASLHRL---G-RHFCGGSLINKEWVLTAAH
Ss_hepsin-like  ---------------SLLCQDCG--RRSLTEDRIVGGVDARQGSWPWQVSLQYD---G-VHQCGGSIISDRWIVSAAH
Ss_matriptase   --------------SDEADCQCG--IRPYRHSRIVGGQASIEGEWPWQVSLHIR---GSSHVCGASVINDRWLVTAAH
                                                :::**  :  .  ** . :           **.::: .  :::***

LsLGST1         CLKEKG--VFVEISRMNVELGVRDARVTSYFTK--RVGV------ESFSIHGEHEEQTAKNDIALVKLKQY-IPFNEG
Ce_Svh-1        CFEEDER-----VSSYEVVVGDWDNNQTDGNEQ--IFYLQRIHFYPLYK-------DIFSHDIAILEIPYPGIEFNEY
Is_Stubble      CVDRTP------ASNLRVRLGEHSIRDTAERYPHEEYTVRRKVVNEGFDR------RNFKNDIALLELSHP-VVFREH
Ss_prostasin    CFSSPS------TSNLVVYVGRQNQKGSNPNEV--NRAVTQIMSHPNYTR------RTNDNDMCLLKLSSP-VTFTNY
Ss_hepsin-like  CFPERN----RQVSRWRVLLGSIYNKLTHKNVR--VLEVKTVVYHSSYLPFVDPNIDDNSRDIAVLALAQP-LHFTDY
Ss_matriptase   CVQDDVKVKYSQPQQWEAYLGLHVQSQTNKWTL--KKNLKQIIQHPGYHA------HTYDNDIALMELDSP-VTLNQN
                *.           .   . :*      :          :        :           ..*:.:: :    : : :

LsLGST1         IKPACLPLDEQQLFAGEWAVASGWGQTSVSDKRG-SNVLKRTRLQVLQNSNQYCINGA--SLGK-PPWIKQMCA--YA
Ce_Svh-1        AQPICLPSKDFVYTPGRQCVVSGWGSMGLRY----AERLQAALIPIINRFD--CVNSS---QIYSSMSRSAFCAGYLE
Is_Stubble      IIPICLPSKGENFTGG-FATVSGWGRLKYGQSY-IPNVLQKVSVEVLENEK--CRTWFKDKGRKEQIYDTMLCAGYKD
Ss_prostasin    IRPVCLAAPGSSFHAGTTSWVTGWGTIRIGVSLPSPKTLQEVDVPVVGNRQ--CNF-N---YGVGSITDNMICAGLAA
Ss_hepsin-like  IQPVCLPHYGQRLIDGQMGTVTGWGNVGYYGTLA--DVLQEANVPIINDAV--CNAPD---YYDNQITTSMFCAGFEK
Ss_matriptase   IWPICLPTATHYFPAGKPVWITGWGTTREGGFEA--SMLQKAEVRIINATV--CNT-----LMEGQTTSNMLCAGVLD
                  * **         *     :***           . *: . : ::      *               . :**

LsLGST1         EGTDSCGGDSGGPLTL----PQNGRCALVGIVSYGLECALTYHAGVYTRVSEYLFWIKHLA-----  256
Ce_Svh-1        GGIDSCQGDSGGPFAC---RREDGAFVLAGVISWGDGCAQKKQPGIYTMVAPYLSWISAIINGQPV  246
Is_Stubble      GGRDSCQGDSGGPLTF----KKNDRVYLIGLVSWGVQCALPSLPGVYTRVSEYVDWVNIYVNN---  264
Ss_prostasin    GGKDSCQGDSGGPMVI----KQGTRWIQSGVVSFGQGCALAKLPGVYARVSQYQTWINSQIS----  263
Ss_hepsin-like  GGT ACQGDSGGPFVAEDSLSKASRYRLLGVVSWGTGCAMAKKPGVYTRVSRFLPWISSAMRTYHN  265
Ss_matriptase   GGVDACQGDSGGPLSS---MEDSGRFFLAGVVSWGDGCARRNKPGVYTQVTKYRDWIKQKTGV---  257
                 * *:*******:       .       *::*:*  **     *:*: *: :  *:.
```

Figure 4. Alignment of *Lepeophtheirus salmonis* labial gland serine protease 1 (LsLGST1) with the trypsin domains of *Caenorhabditis elegans* svh-1 (AB693146), *Ixodes scapularis* serine proteinase stubble (XM_042288799) and from *Salmo salar* serine protease hepsin-like (XM_014201488), ST14 transmembrane serine protease matriptase b (XM_014128968) and prostasin (XM_014193631). The activation cleavage site (IVGG) is underlined and marked in green, where the preceding R/K is in bold where cleavage is executed between the R/K and the I. All sequences lack the enteropeptidase consensus sequence (DDDK/R), though a single D is seen in *Salmo salar* hepsin-like gene. The active site triad residues, H, D and S which catalyze the hydrolysis of amide bonds are marked in gray. While the key residues in the specificity pocket, D, G and G, are marked in black.

Apyrase

After RACE, the LsLGAp1 mRNA was found to be 1836 nt having an ORF of 1671 nt encoding 556 aa’s. A signal peptide was identified N-terminally, and preceding this signal peptide, a 5'-Nucleotidase/apyrase domain was predicted. The apyrase domain contained an N-terminal metal ion binding and catalysis domain and a C-terminal nucleotide substrate binding domain identified (Fig. 5). Within the N-terminal domain, aspartic acid, asparagine and histidine residues known to take part in binding the two zinc-ions needed for catalysis were found to be conserved in LsLGAp1, corresponding to D^{29}, H^{31}, D^{79}, N^{111}, H^{211}, H^{234} and H^{236} (Heuts, Weissenborn et al. 2012, Knapp, Zebisch et al. 2012). Also, the histidine and the aspartate that forms a catalytic Asp-His-dyad that stabilizes the transition state is found, so where cysteines known to be involved in intramolecular disulfide bridge formation. Such cysteines were, however, not found to be conserved within the C-terminal substrate binding domain. Here, cysteines in human CD73 are probably important to stabilize a conserved loop involved in homodimerization by the means of hydrogen bonds and hydrophobic interactions (Heuts, Weissenborn et al. 2012). Though, some residues found to be involved in this non-covalent dimerization are found in LsLGAp1 (D^{356}, P^{456} and D^{520}), but as the dimerization interface of human CD73 is connected through a network of hydrophobic interactions and hydrogen bonds (Heuts, Weissenborn et al. 2012, Knapp, Zebisch et al. 2012), it is difficult to extrapolate this to the potential of LsLGAp1 dimer formation. The C-terminal domain is also involved in substrate binding, where phenylalanine residues is found to sandwich the nucleobase in adenosine (Knapp, Zebisch et al. 2012). The two phenylalanine is seen as tyrosine residues in LsLGAp1, that also is an aromatic residue that can allow strong hydrogen bonds to form with an adenosine base. Moreover, charged residues important in binding the ribose moiety is found. No potential N-linked glycosylation or GPI-modification sites were found, while three possible O-linked glycosylation sites were found with a weak probability.

BlastP searches (NCBI) with LsLGAp1 showed the highest resemblance to genes annotated as snake venom 5'-nucleotidase in various daphnia species, followed by various 5'-nucleotidases from crustacean, hexapod and chelicerate species. None of the apyrases from *C. elegans* was found to be similar to LsLGAp1 when limiting the Blast search to this species, while multiple 5'-nucleotidase-like genes or genes annotated as ecto-5'-nucleotidase were retrieved from *D. melanogaster*, *I. scapularis* and *A. aegypt.* Limiting the blast search to vertebrate species, LsLGAp1 was found to have the highest resemblance to fish apyrases annotated as snake

venom 5'-nucleotidase and ecto-5'-nucleotidase, or CD73, with 37 % identity and 56 % similarity to human CD73.

```
sLGAp1        MSIL----------IQGLV-------FYILLEIVTPFQLNIIHINDIHVRMEEIDSRGETCSASS---SRCFGGLARVKY
Is5NUC-like   MSI------FRFVIFETLL-------FIGGASNENGFILTVIHTNDIHSHFEESNKGGGSCSEKNKRAKECFGGVARILT
AgApyrase     MEM---ARLTAMVVLLAMCHAGLDAATVIDRKRDVLFPLTVIHWNDFHARFEETNVLSTRCDVEA--GEKCIGGYARVVS
Dp5NUC-like   MRTLSTRNALPLLWLF--L-------FVVHWT-SASFNLTILHTNDIHCRFDEANKNAGTCRQQDSDKGNCFGGYARLVH
SsCD73        MGVS--ESMKCLLFYL--C-------ICSHVSTVDAWELTLLHTNDVHARVEETSNSYGKCTK-----PPCFAGVARRFT
HsCD73        MCPR-AARAPATLLLA--L-------GAVLWPAAGAWELTILHTNDVHSRLEQTSEDSSKCVN----ASRCMGGVARLFT
              *                                           : *.::* **.* :.:: .      *         *:.* **

LsLGAp1       LADNMKR--SLHNPLFLNGGDFYQGTNWYSFYKWKAVTHFSNQLGFDAMALGNHEFDDGNKGLK-PFLEG---AKFPIIC
Is5NUC-like   KVKELKA--RSENPLFFNAGDFYQGTVWYTVHRHKIVSEAMSRMMYDAVCLGNHEFDDGPEGLV-PFLKKMEAANVAVLG
AgApyrase     AVNSLSEQYKERNPVFLNAGDNFQGTFWYTLLKWNVTSYFLNLLPADAVTLGNHEFDHGVAGVV-PFMET---LKSPIVV
Dp5NUC-like   QARQIRN--QHPNTIFLNAGDFFQGTIWYSIHKWKAVSHFGNMLNLTAMSLGNHEFDDGVDGLI-PFVES---ANHPVLA
SsCD73        KIQEIRS--REKNVMLLDAGDQFQGTVWFNVYKGAEAAYFMNKLGYDAMALGNHEFDNNVEGLIKPFLQE---VKCTVLS
HsCD73        KVQQIRR--AEPNVLLLDAGDQYQGTIWFTVYKGAEVAHFMNALRYDAMALGNHEFDNGVEGLIEPLLKE---AKFPILS
                .:       * ::::.** :*** *:.. :    .:    . :    *: *******..  *:   *:::      :  ::

              N-terminal metallophosphatase domain
LsLGAp1       ANIDASK--DPILRNK-FQKSITINVGGKRIGIVGYVTLRTEYTSQPDDTLVFTEEVEAITKEAKNLKGKGVDIIIALGH
Is5NUC-like   TNLETSE--EPRMKEIRLRRSIVYSVQGQKIGVMGVVTTETRTIARPGRVKIF-DEIISIRAEVKRLRQKGVRIFILISH
AgApyrase     ANIDDSE--EPMMQGK-YNKSVVIKRGERQIGIIGVIHHSTDTLSMTE-RLRFLDEIEAINTEAERLKASGVNIIIVLSH
Dp5NUC-like   ANIDSSN--EPRLQGK-ISKSIVVQVGGKSVGIIGYLTTETTYISSAGNVKIF-DEVQGVRDEAERLKKSGVNILIAVGH
SsCD73        ANIKADDTLAPHISGL-YFPYKIFDVGSQKVGVVGYTSKETPALSQTGPNLVFEDEIDALQPQVDKLITLGVNKIIALGH
HsCD73        ANIKAKGPLASQISGL-YLPYKVLPVGDEVVGIVGYTSKETPFLSNPGTNLVFEDEITALQPEVDKLKTLNVNKIIALGH
              :*:. .       :                . :*::*      *    :         * :*: .:  :...*    .*   :* :.*

LsLGAp1       SGYDKDQEIARKIPEIDVVVGGHSHSFLYHTNNGASQSGDYIEGDYPTMVRQPSGKQVPVVQAFAFTKYLGKLTVNFDIN
Is5NUC-like   VGYAKDKEIAIAIPELQLIVGGHSNTFLYSGRS--PRKEDVIEGPYPTVVKRGDGSFALVVQGFWFGKYLGHLKLRFSND
AgApyrase     CGITIDKKIAAQCADVDIIVGGHSHTFLYNGSA--EGFPDAAVDTYPVVIQQTSGRKVPVVQASCFTKFVGRLTAYFDEA
Dp5NUC-like   AGYLKDMEIAAKVPDIDVVVGGHTNTFLWNGPA--PSIE-EPQGPYPTMVKQPSGKSVPVVQAFAFGKYLGNLMVTFNDE
SsCD73        SGFVTDKEIAKRVKGVDVVIGGHSNTFLYTGEK--PSSE-VPAGPYPVMVKSEDGRDVPVVQAFAFGKYLGYLKVTFDEA
HsCD73        SGFEMDKLIAQKVRGVDVVVGGHSNTFLYTGNP--PSKE-VPAGKYPFIVTSDDGRKVPVVQAYAFGKYLGYLKIEFDER
               *    *  **      :::::***:::**:                 . ** ::    .*   . ***.  * *::* *    *.

LsLGAp1       NRVTSAYGNPVLLDQNVREDVGMKSELKEWKKSITAFEKSVVGVASVDLI--PSQSR--ESNLGNVVTDSMLYGLRSK--
Is5NUC-like   GRLTGWDGNPILLDPSIQEDRLMIKMLTVYKKQVEKASKECIGFTKVLLEASHKVCRLKECNMANLIADAFLAHYANRKS
AgApyrase     GNLVEWEGNPVYLDESIPKDPRILQEMVPWREQVDVLAYRNVGSSLVVLS--KAECRTGECNFGSFVADAFVDYYTQRP-
Dp5NUC-like   GEVIATAGLPILMDKSIPQDPSVLQELIPFRKEVEALSEKEVGKTRVFLDGNRLSCRMVECNLGNFLADAYVDLFTKFA-
SsCD73        GNVVQSTGNPILLDSSVPEDPSILADVNEWKKALANYSSQYVGETLVYLNGTFEECRFRECNLGNLICDAMVHHNIKYA-
HsCD73        GNVISSHGNPILLNSSIPEDPSIKADINKWRIKLDNYSTQELGKTIVYLDGSSQSCRFRECNMGNLICDAMINNNLRHA-
              ..:    * *: :: .: :*  :     :    :         :* : * *       .*   *.*:...: *: :

LsLGAp1       ------YDVRLAIFNSGSLRSTIN----AGRVTQGDILKAIPYGNPNYIVKLSGRSLINIFELWVNG--------GRGFL
Is5NUC-like   AIPHAWSDVNAAVVNAGITKWSIP----QGAIRRENMMSAMPFESTLVVLTMSGAQLWKMFDFSVSQFTWY-DDPVGAFL
AgApyrase     ENSNEWTYASIGITNDGGMRTSLK----KGTLTYDDIVTAVPYENTVDTFDIRGQYLLDALEYSASRFNSA------DVL
Dp5NUC-like   -GEGQWNKVAIALVNSGGIRASIDERAQNGSITYGDLLAVAPFSNTVDIIKISGETLKNIFEFTVHDYDPKALDPFGGFL
SsCD73        -DELQWNHVSSCIIQGGSIRSSIDERSRNGSITMEDLITVLPFGGTYDLVQLKGSTLRKAFEHSVRRYGGN----TGEFL
HsCD73        -DETFWNHVSMCILNGGGIRSPIDERN-NGTITWENLAAVLPFGGTFDLVQLKGSTLKKAFEHSVHRYGQS----TGEFL
                         .    : : *   :   :        * :     :: . *: .        . : *   *  . ::   .          .*

              C-terminal 5'nucleotidase domain
LsLGAp1       QVSGFKVFIDKTRRGGFLVKVLSVSRHN--PRMYCPLKKYKLYPVAIPSYIAGGGERYSIIPKKEHGIKKDTQTDQELLA
Is5NUC-like   QVSGIKVAYNFARCAFQRVIRLEILCANCSIPKYEPVQWKNTYKIVTTSYIANGGDGFNFDTDL-KK-ETEGAIDNEVVT
AgApyrase     QVSGIRVTYNITRSAGNRVVSVDIRCRECKVPRYEPLDRNKYYRVAIAAWIGNGGNGYTMFGEHRTN-PRIGPLDIVVLE
Dp5NUC-like   QVAGVRVVYDISRSKGDRVMELLARCNNCRIPIYRPVQPEETYDIAVASFAGGGDGFAIKGNIIEH-TLTGSLDVDTII
SsCD73        QVSGFHVEYDMSRPPGERARSISVLCTNCRVPVYEPLDDSRLYKVVLPSYLVEGGDGFSMIKEEKLK-HDSGDMDISVVA
HsCD73        QVGGIHVVYDLSRKPGDRVVKLDVLCTKCRVPSYDPLKMDEVYKVILPNFLANGGDGFQMIKDELLR-HDSGDQDINVVS
              **.*.:*   : :*         .  :      :      * *:.   . * :     ::   **: : :   .                *   :

LsLGAp1       NYFKAKSPISMSKRDGRILIHHFRSASNEFRSNPYWKTLYDNWNNCAIYPS---------------------  556
Is5NUC-like   QYVRKMSPIKQ-AEEGRVVMYNNERPAGAT-----SGGLYQAYQS-QG------------------------  573
AgApyrase     QYVAKMSPIMQ-GTDGRIRVVS--------------------------------------------------  552
Dp5NUC-like   GYMERISPITT-GVEGRIRFANETNLMDCGKT---KNNANSGSTNLSGFATSAVPISSLTALLLLLSQTMIK  602
SsCD73        SYITERKKVHP-AVEGRIKISNSNSG---G-----------------SGHTT---LL-ILIGLVIALSRSL--  574
HsCD73        TYISKMKVIYP-AVEGRIKFSTGSHC---H-----------------GSFSL---IFLSLWAVIFVLYQ----  574
               *.    . :      :**: .
```

Figure 5. *Lepeophtheirus salmonis* labial gland apyrase 1 (LsLGAp1) aligned with an *Aedes aegypti* apyrase (XM_001648629), *Ixodes scapularis* 5'-nucleotidase-like protein (XM_029966494), *Daphnia pulex* snake venom 5'-nucleotidase-like protein (XM_046593699) and CD73 from *Salmo salar* (XM_014204939) and *Homo sapiens* (BC065937). The N-terminal metal ion binding and catalysis domain and the C-terminal substrate binding domain are indicated above the sequences in dark and light grey, respectively. Residues taking part in binding the two zinc-ions are marked in dark grey. The histidine and the aspartate that forms a catalytic Asp-His-dyad is marked in black. Cysteines known to be involved in intramolecular disulfide bridge formation are marked in blue, while residues found to be involved in non-covalent dimerization are marked in green. The aromatic and charged residues in the C-terminal domain found to interact with the substrate are underlined and marked in black and grey, respectively.

References

Gomis-Rüth, F. X. and Stöcker, W. (2023). Structural and evolutionary insights into astacin metallopeptidases. *Front Mol Biosci* 4: 9:1080836. doi: 10.3389/fmolb.2022.1080836.

Heuts, D. P., M. J. Weissenborn, R. V. Olkhov, A. M. Shaw, J. Gummadova, C. Levy and N. S. Scrutton (2012). Crystal structure of a soluble form of human CD73 with ecto-5'-nucleotidase activity. *Chembiochem* 13(16): 2384-2391. doi: 10.1002/cbic.201200426.

Honma, T. and K. Shiomi (2006). Peptide toxins in sea anemones: Structural and functional aspects. *Marine Biotechnology* 8(1): 1-10. doi: 10.1007/s10126-005-5093-2.

Knapp, K., M. Zebisch, J. Pippel, A. El-Tayeb, C. E. Muller and N. Strater (2012). Crystal structure of the human ecto-5'-nucleotidase (CD73): insights into the regulation of purinergic signaling. *Structure* 20(12): 2161-2173. doi: 10.1016/j.str.2012.10.001.

Muhlia-Almazan, A., A. Sanchez-Paz and F. L. Garcia-Carreno (2008). Invertebrate trypsins: a review. *J Comp Physiol B* 178(6): 655-672. doi: 10.1007/s00360-008-0263-y.

Perona, J. J. and Craik, C. S. (1995). Structural basis of substrate-specificity in the serine proteases. *Protein Science* 4(3): 337-360. doi: 10.1126/science.3838593

Øvergård, A. C., Hamre, L. A., Harasimczuk, E., Dalvin, S., Nilsen, F. and Grotmol, S. (2016). Exocrine glands of *Lepeophtheirus salmonis* (Copepoda: Caligidae): Distribution, developmental appearance, and site of secretion. *J Morphol* 277(12): 1616-1630. doi: org/10.1002/jmor.20611.
