# Supplementary figures and images for: Salmon louse labial gland enzymes: implications for host settlement and immune modulation
Source: Front Genet. 2024 Jan 17;14:1303898. doi: 10.3389/fgene.2023.1303898 (PMC10828956; doi:10.3389/fgene.2023.1303898)

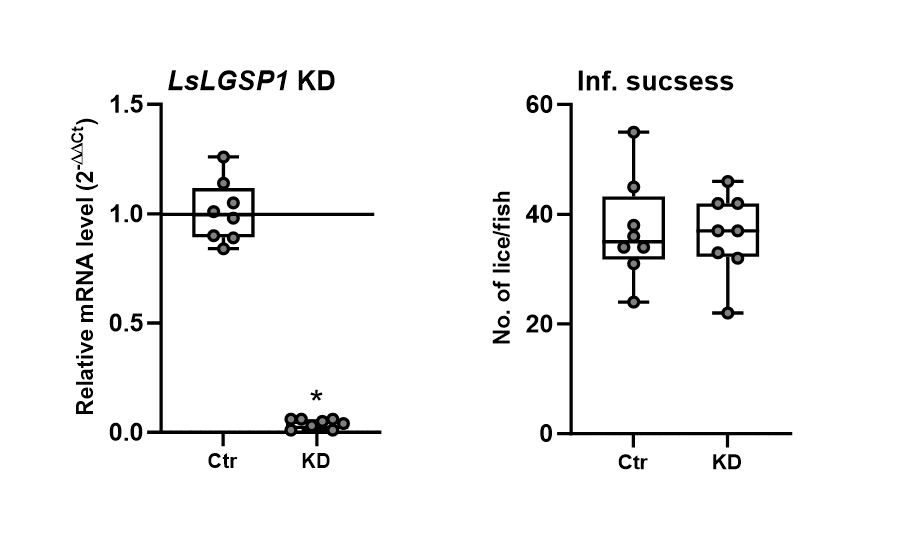

Supplement: Supplementary file 2 [file Image1.TIF]
