# Supplementary material for: Salmon louse labial gland enzymes: implications for host settlement and immune modulation
Source: Front Genet. 2024 Jan 17;14:1303898. doi: 10.3389/fgene.2023.1303898 (PMC10828956; doi:10.3389/fgene.2023.1303898)
Supplement: Supplementary file 3 [file DataSheet1.PDF]

Table 1: Sequences of primers used for characterization of the labial gland enzymes. Primers were used in rapid amplification of cDNA ends (RACE), confirming the open reading frame (ORF), synthesis of RNA probes for in situ hybridization (IS) and double stranded RNA for RNA interference (RNAi), and for real time RT-PCR both target genes (qPCR) and reference genes (qPCR ref). Gene name abbreviations: Lepeophtheirus salmonis (Ls), labial gland protein (LGP), labial gland astacin (LGA), labial gland serine protease 1 (LGSP1), labial gland apyrase (LsLGAp1), Trypsin 1 (Tryp1), elongation factor (EF), adenine nucleotide translocator (ADT), Salmo salar (Ss), interleukin (IL), matrix metalloproteinase 13 (MMP13), tumor necrosis factor alpha (TNFα), non-spesific cytotoxic cell reseptor P1 (NCCRP1), interferon gamma (IFNγ), T-cell reseptor beta (TCRβ), cluster of differentiation (CD), major histocompatibility complex II (MHC2), immunoglobulin (Ig), tripartatie motif-containing protein 16 (TRIM16).

**Gene**	**Forward**	**Reverse**	**Usage**
LsLGP1L	AAACAATGGCTATAAAATGAATAATCCCATT	GAATAACCTCGTCATTCAGAATAACACCCT	qPCR
LsLGP1	AAAACGAAGAGACAGTTGTCCCAAATT	CGTCATTCAGAATAACATCGTTTTTCTC	qPCR
LsLGP2	GCTCTCCTTAGTATCTTTATTTTGGGCTCC	ACACGTAAGCACAGCTTGTGGATGC	qPCR
LsLGP3	TCAAGAAGGATTTGAAAAGTTGACGAATG	TGCCCGATTGTTCCATAATTCCGTT	qPCR
LsLGP4	TGTTTTTTCAAGATCGACTCTTCCAATGG	AGCACCTTTCTTTTTTGCAAGTTCCATA	qPCR
LsLGSP1	GGGACAAACCTCGGTCAGCGAT	CGCACATTTGCTTTATCCATGGTGGTTTCCCA	RACE
LsLGSP1	TCAGTAAAAGCTGTTGTAGGTGAACG	ATTCCACATATGCCCACAAGGAT	ORF
LsLGSP1	GGGACAAACCTCGGTCAGCGAT	CCTGGTCGCACTTAACTTCATGCC	IS/RNAi
LsLGSP1	TCTTAATACTGTTATGCCTGGGAGTCTTGG	TGCATTGGGATCAATTTCAGCAATT	qPCR
LsLGA1	TCCATTATACAAAAATCAATGCAAGACTAA	ATCTTTGATAAATTGGTTAGTCTTGCATTGA	RACE
LsLGA1	GGCAAGTCCACAGCTGAGCATTGCG	TCTACAAGTTAATTTACATCCTTTTGTAATGGC	ORF
LsLGA1	GACTTGGATGTGATTAAATC	GCATAATAGATCCATAGTCA	IS/RNAi
LsLGA1	CTCACTTTCCATGATGTCTATTCAAGCG	CCTTGAAGCCATTTCCTGTCTGTGA	qPCR
LsLGA2	ATTAACAAGTTATTGCGAAAGCCGGCCAGA	CATTCTCCCCACAAAGATGGCCCAATTCGG	RACE
LsLGA2	CAAGCGTTCGTATTTGACCGTGGCA	TCCACACGTTAATTTACAACTATTTGTAATTTC	ORF
LsLGA2	GAAGTGAGTGTGATTAAATC	GCATGATGGATCCATAGTCA	IS/RNAi
LsLGA2	CTCCTGACTCTTCATGATGTCTATTCAAGTG	TGGAAGCCATTTCCTGCCTGTGA	qPCR
LsLGA3	CAAGTTATTGCCAAAGCAATCCATCAGTCA	CATTCTCCCCACAAAGATGGCCCAAATCGG	RACE
LsLGA3	CGCCATTCCGCAGCTGAGCATTGCA	TCTACAAGTAAGTTTACAGCCTTCTGTAACTCC	ORF
LsLGA3	GATGTGGATGTGATTAAATC	GCATGATAGATCCATAGTCA	IS/RNAi
LsLGA3	CCTGACTTTCCAGAATGTCTATTCAAGCG	ATTCCTTGAAGCAATTTCCTGTCTATGG	qPCR
LsLGA4	TTTATACAAAAAATATTGCGAAAGCCATCA	AACCTTACAACCATCTTTGATAGATTGATG	RACE
LsLGA4	CAAAAGTCCACAGCTGAACATTGCA	TCTACAAGTTAATTTACAGTCTTTTGTAATGGT	ORF
LsLGA4	GATGTGGATGTGATTAAATC	GCATGATAGATCCATAGTCA	IS/RNAi
LsLGA4	CCTGACTTTCCATGATGTTTATTCAAGCA	AAATTTCTTGAAGCCATTTCCTGTTTGTAA	qPCR
LsLGA5	TGATCGATGTTGGTCTGGATACCG	CCGTTGATTAGTTCGTGGGATCCAT	RACE
LsLGA5	CCTTTGGATTTGCATATGACTACC	AGACATACTTTCGATGGCCTTCAT	RACEn
LsLGA5	CATCAGTTGAATAACAAAAATGATGA	AATTACAATGACTTCAGTTATCACAAA	ORF
LsLGA5	CGGCAAGACCTTGCTTCTAGGAAT	TTTGTGCTCATCGTAGGGTATCCC	IS/RNAi

Gene	Forward	Reverse	Usage
LsLGA6	GCACCTACTACCACAACCCCAGCAT	GCCAATATTAGCGAAGCATCCGTCT	RACE
LsLGA6	AGCGACTATAAGCATCTTTGTGGAA	ATGTTCGATCCGAAATGTGCTTA	RACEn
LsLGA6	TCATTAGGTTTTTAAAGATGGCAAA	TTTTCTTGATTGCTCCAACGAA	ORF
LsLGA6	ATGCGTCACATATGTTGAAAGAACA	CCACAAAGATGCTTATAGTCGCTACA	IS/RNAi
LsLGA6	GAATGAACCTGCTGCTATTCCA	TGAAAAGTATCGAAGTCCGTAGTGC	qPCR
LsLGA7	GGTCATGAGCAAACACGACCTGACCG	CTGGGTCTGCATTGGGGTGGTACGAT	RACE
LsLGA7		TAATACCACTATCGTACCACCCCAATGCAG	RACEn
LsLGA7	AGCATATTCCGAACTGAGCCAT	CCACAAGTGGCTTTACAATTCTC	ORF
LsLGA7	TTCCGAACTGAGCCATGCTCAA	CGAATTCCAAACGGTACAGGTCTCAG	IS/RNAi
LsLGA7	ATGGTGCCCCTTTAAATATACTAGC	GTCAAAGCAATCTGAAGTGGTTGTA	qPCR
LsLGA8	CCAAAAAATGTGATCCATCCAGA	CCATGTTTCCCCTCCTTGTT	IS/RNAi
LsLGSP1	TCTTAATACTGTTATGCCTGGGAGTCTTGG	CGCACATTTGCTTTATCCATGGTGGTTTCCCA	RACE
LsLGSP1	TCAGTAAAAGCTGTTGTAGGTGAACG	ATTCCACATATGCCCACAAGGAT	ORF
LsLGSP1	GGGACAAACCTCGGTCAGCGAT	CCTGGTCGCACTTAACTTCATGCC	IS/RNAi
LsLGSP1	TCTTAATACTGTTATGCCTGGGAGTCTTGG	TGCATTGGGATCAATTTCAGCAATT	qPCR
LsLGAp1	TGTGAAACTCTCTGGACGCTCACTCATCAA	TGTGTCGTCGGGTTGTGAGGTGTACTCAG	RACE
LsLGAp1	ATAGTCACGCCTTTTCAGCTA	GATCCGGCCATCCCGTTTAG	ORF
LsLGSP1	TCTTAATACTGTTATGCCTGGGAGTCTTGG	TGCATTGGGATCAATTTCAGCAATT	qPCR
LsLGAp1	TGTGAAACTCTCTGGACGCTCACTCATCAA	TGTGTCGTCGGGTTGTGAGGTGTACTCAG	RACE
LsLGAp1	ATAGTCACGCCTTTTCAGCTA	GATCCGGCCATCCCGTTTAG	ORF
LsLGAp1	AAATGGAAGGCAGTGACGCAT	GACCTCCGACAACGACATCA	IS/RNAi
LsLGAp1	ATAGTCACGCCTTTTCAGCTA	GCATCTCGAAGAAGAAGCG	qPCR
LsTryp1	CACCTTCTCCAGTTCTTAAAGCTGTT	AGATCATGGTCTCATCAATAGATCCA	qPCR
LsEF1α	GGTCGACAGACGTACTGGTAAATCC	TGCGGCCTTGGTGGTGGTTC	qPCRref (ref)
LsADT3	CTGGAGAGGGAATTTGGCTAACGTG	GACCCTGGACACCGTCAGACTTCA	qPCRref (ref)
SsIL1β	GCTGGAGAGTGCTGTGGAAGA	TGCTTCCCTCCTGCTCGTAG	qPCR
SsIL8	GCATCAGAATGTCAGCCAGCC	ACGCCTCTCAGACTCATCCC	qPCR
SsIL6	ACCAACAGTTTGTGGAGGAGTTTCAGAAGC	CCTGCAGACATGCCTCCTTGTTG	qPCR
SsMMP13	ACTCTTTGCCAATATCGCCACCCA	TGGGCCCTCGTTTGAACGCA	qPCR
SsTNFα	CACTGCCACCAAGAGCCAAG	CGCCAGTTGTCATCGCATACC	qPCR
SsNCCRP1	AATCCTGCGCCTCACGGTGTGAGTC	GCGAGGAGGTCCTTCTGGTGGAAAC	qPCR
SsIL10	ATGAGGCTAATGACGAGCTGGAGA	GGTGTAGAATGCCTTCGTCCAACA	qPCR
SsIFNγ	ATGGATGTGTTATCAAGGGCTGTGATGTG	CAGCTGGTCCTTGGAGATCTTATAGTGGAC	qPCR
SsIL4/13a	CGTACCGGCAGCATAAAAATCACCATTCC	CCTTGCATTTTGTGGTGGTCCCA	qPCR
SsMHCII	GGACGTGAGGTGAAGTCTGATGTGACC	CTGATGTGCTCCACCATGCAGGA	qPCR
SsIgM	TGAGGAGAACTGTGGGCTACACT	TGTTAATGACCACTGAATGTGCAT	qPCR
SsIgT	GGTGGTCATGGACGTACTATTT	CCTGTGCAGGCTCATATCTT	qPCR
SsIgD	CACCAGGAGGAAAGTTTGGCATCA	CCCCAAGGAGCTCTGGTTTGGA	qPCR

Gene	Forward	Reverse	Usage
SsCD4	GAGTACACCTGCGCTGTGGAAT	GGTTGACCTCCTGACCTACAAAGG	qPCR
SsCD8α	TAGAGTGCAAGACAACGCTGGAATGGA	TCTCGAGCCTTTTTGAAAGCCTTCAG	qPCR
SsEF1α	CACCACCGGCCATCTGATCTACAA	TCAGCAGCCTCCTTCTCGAACTTC	qPCR (ref)
SsTRIM16	TTACTGTAGGAGCTGTATTGAGGGCTGCTG	TTCTCCACCAGCTCAGCCAACATG	qPCR (ref)

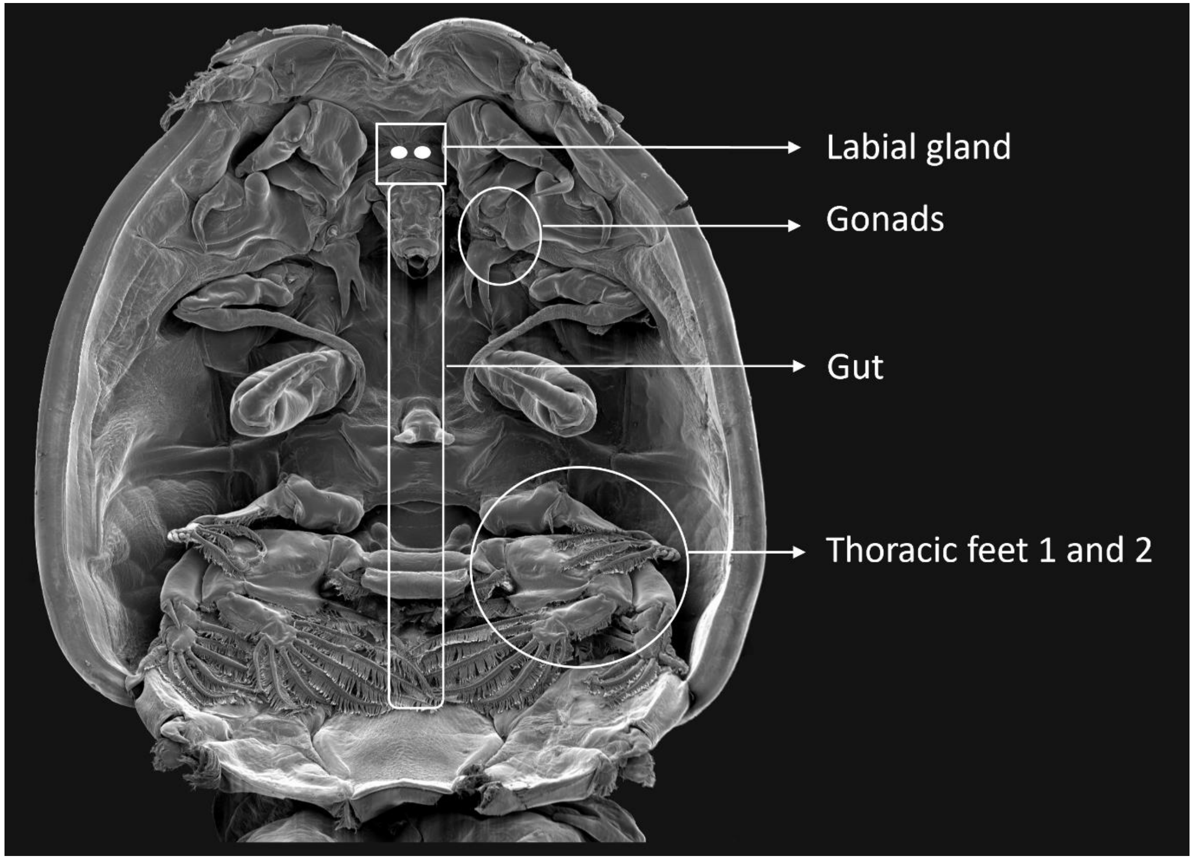

Figure 1. An overview of where the sample high in labial gland were dissected from, and the other areas used to compare the transcriptome of the labial gland sample to enable the identification of labial gland expressed genes. If the genes were high in the labial gland sample, but not in gut, gonads and the thoracic feet that holds all the other gland types identified in the lice, the gene were analyzed further if it was mainly expressed in parasitic lice stages.
